# Shikonin blocks CAF-induced TNBC metastasis by suppressing mitochondrial biogenesis through GSK-3β/NEDD4-1 mediated phosphorylation-dependent degradation of PGC-1α

**DOI:** 10.1186/s13046-024-03101-z

**Published:** 2024-06-27

**Authors:** Shuangqin Fan, Xiaomin Yan, Xiaoxia Hu, Xing Liu, Shijie Zhao, Yue Zhang, Xiaofeng Zhou, Xiangchun Shen, Qi Qi, Yan Chen

**Affiliations:** 1 The High Efficacy Application of Natural Medicinal Resources Engineering Center of Guizhou Province, School of Pharmaceutical Sciences, Guizhou Medical University, No.6 Ankang Avenue, Guian New District, Guizhou, 561113 China; 2 Key Laboratory of Novel Anti-Cancer Drug Targets Discovery and Application, School of Pharmaceutical Sciences, Guizhou Medical University, Guizhou, 561113 China; 3 The Key Laboratory of Optimal Utilization of Natural Medicine Resources, School of Pharmaceutical Sciences, Guizhou Medical University, No.6 Ankang Avenue, Guian New District, Guizhou, 561113 China; 4 State Key Laboratory of Bioactive Molecules and Druggability Assessment, MOE Key Laboratory of Tumor Molecular Biology, Guangdong Province Key Laboratory of Pharmacodynamic Constituents of TCM and New Drugs Research, Department of Pharmacology, School of Medicine, Jinan University, Guangzhou, 510632 China

**Keywords:** Mitochondrial biogenesis, Metastasis, Peroxisome-proliferator activated receptor coactivator 1α, Cancer-associated fibroblast, Triple-negative breast cancer

## Abstract

**Background:**

Triple-negative breast cancer (TNBC) is characterized by its high metastatic potential, which results in poor patient survival. Cancer-associated fibroblasts (CAFs) are crucial in facilitating TNBC metastasis via induction of mitochondrial biogenesis. However, how to inhibit CAF-conferred mitochondrial biogenesis is still needed to explore.

**Methods:**

We investigated metastasis using wound healing and cell invasion assays, 3D-culture, anoikis detection, and NOD/SCID mice. Mitochondrial biogenesis was detected by MitoTracker green FM staining, quantification of mitochondrial DNA levels, and blue-native polyacrylamide gel electrophoresis. The expression, transcription, and phosphorylation of peroxisome-proliferator activated receptor coactivator 1α (PGC-1α) were detected by western blotting, chromatin immunoprecipitation, dual-luciferase reporter assay, quantitative polymerase chain reaction, immunoprecipitation, and liquid chromatography-tandem mass spectrometry. The prognostic role of PGC-1α in TNBC was evaluated using the Kaplan–Meier plotter database and clinical breast cancer tissue samples.

**Results:**

We demonstrated that PGC-1α indicated lymph node metastasis, tumor thrombus formation, and poor survival in TNBC patients, and it was induced by CAFs, which functioned as an inducer of mitochondrial biogenesis and metastasis in TNBC. Shikonin impeded the CAF-induced PGC-1α expression, nuclear localization, and interaction with estrogen-related receptor alpha (ERRα), thereby inhibiting PGC-1α/ERRα-targeted mitochondrial genes. Mechanistically, the downregulation of PGC-1α was mediated by synthase kinase 3β-induced phosphorylation of PGC-1α at Thr295, which associated with neural precursor cell expressed developmentally downregulated 4e1 recognition and subsequent degradation by ubiquitin proteolysis. Mutation of PGC-1α at Thr295 negated the suppressive effects of shikonin on CAF-stimulated TNBC mitochondrial biogenesis and metastasis in vitro and in vivo.

**Conclusions:**

Our findings indicate that PGC-1α is a viable target for blocking TNBC metastasis by disrupting mitochondrial biogenesis, and that shikonin merits potential for treatment of TNBC metastasis as an inhibitor of mitochondrial biogenesis through targeting PGC-1α.

**Supplementary Information:**

The online version contains supplementary material available at 10.1186/s13046-024-03101-z.

## Introduction

Triple-negative breast cancer (TNBC) is widely recognized as the most aggressive subtype of breast cancer, characterized by high metastatic risk and low overall survival [1]. Owing to the absence of specific molecular targets, TNBC is unresponsive to endocrine therapy or anti-human epidermal growth factor receptor 2-targeted therapy [2]. Current clinical trials are investigating new treatment for TNBC, including targeted therapies such as cyclin-dependent kinase 4/6 inhibitors and poly ADP-ribose polymerase inhibitors, alongside immunotherapies [3]. Despite chemotherapy being the mainstay of systemic treatment, it often results in unsatisfactory outcomes due to high recurrence rates and poor prognosis [4], highlighting the critical need for novel therapeutic strategies and agents to combat TNBC. Research has shown that during metastasis, TNBC cells increasingly rely on mitochondrial metabolism [5]. Invasive TNBC cells display enhanced mitochondrial biogenesis, oxidative phosphorylation (OXPHOS), and ATP production when colonizing distant organs [6]. Consequently, targeting mitochondrial biogenesis and metabolism has emerged as an effective approach to limit TNBC metastasis [6], offering a potential avenue to address treatment challenges in TNBC.

Cancer-associated fibroblasts (CAFs) are key stromal cells within the tumor microenvironment (TME), playing vital roles in supporting tumor growth, remodeling the extracellular matrix, creating metastatic niches, and shielding tumor cells from the immune system, which are significant contributors to tumor metastasis [7, 8]. Specifically, in TNBC, CAFs engage in metabolic symbiosis with cancer cells, referred to as the “reverse Warburg effect” [9], which is linked to poor clinical outcomes [10]. In this process, cancer cells prompt mitochondrial dysfunction and a shift towards aerobic glycolysis in CAFs. As a result, CAFs generate energy-rich products, such as L-lactate, glutamine, ketones, and fatty acids, which then supply cancer cells for mitochondrial oxidative phosphorylation (OXPHOS) [11]. Additionally, metabolites from glycolytic CAFs drive mitochondrial biogenesis and activity in breast cancer cells by boosting mitochondrial proteins and chaperones, thus enhancing their anabolic capabilities, biomass, and invasiveness [12]. Therefore, CAFs play indispensable roles in mitochondrial biogenesis in breast cancer cells, facilitating their metastatic potential.

Peroxisome proliferator-activated receptor coactivator 1α (PGC-1α) serves as a central regulator of mitochondrial biogenesis and energy sensing [13, 14]. As a transcriptional coactivator, PGC-1α partners with nuclear transcription factors to bind promoters and activate transcription of their target genes [15]. Among these factors, estrogen-related receptor alpha (ERRα) is a key modulator of cellular metabolism in breast cancer [16]. In TNBC tissues, elevated nuclear ERRα expression has been associated with shorter survival and recurrence-free survival (RFS) in TNBC patients [17]. ERRα regulates genes involved in mitochondrial proteins and enzymes crucial for the tricarboxylic acid (TCA) cycle and OXPHOS pathways in TNBC cells [18, 19]. Therefore, the PGC-1α/ERRα axis may play a pivotal role in mitochondrial biogenesis in TNBC cells.

Shikonin, a bioactive compound extracted from the roots of *Lithospermum erythrorhizon Siebold* & *Zucc*., has demonstrated a spectrum of pharmacological properties, including anti-oxidative stress, anti-inflammatory, anti-bacterial, anti-viral, and immunomodulatory effects [20]. Moreover, shikonin has shown potent inhibitory effects against multiple types of cancer [21, 22]. However, its impact on the TME, particularly on CAF-stimulated mitochondrial biogenesis and metastasis in TNBC, remains unexplored. In the present study, we investigate the effects of shikonin on CAF-stimulated metastasis of TNBC cells in vitro and in vivo, and explored the underlying mechanisms on the inhibition of mitochondrial biogenesis. Our results suggested that shikonin is a promising candidate as an inhibitor of CAF-induced mitochondrial biogenesis and TNBC metastasis treatment, which offers new insights of mitochondrial biogenesis as a target for developing drug against TNBC.

## Materials and methods

### Materials

Detailed information regarding the materials is provided in the supplementary material.

### Cell culture

MDA-MB-231, MDA-MB-468, MCF-7, T-47D, SK-BR-3 (Chinese Academy of Sciences, Kunming, Yunnan, China), MCF-10A, MCF-12A, and WI-38 cell lines (American Type Culture Collection, Manassas, VA, USA), and immortalized primary CAFs from TNBC tissues (BeNa Culture Collection, Beijing, China) were cultured as described in the Supplementary Material. The patient provided consent for use of the tissue and had not received any prior therapy. All cell lines were tested for mycoplasma contamination and authenticated using short tandem repeat profiling. CAFs were identified using the CAF-related biomarkers alpha-smooth muscle actin (α-SMA) and fibronectin, confirming a purity of > 99% (Fig. S1) and utilized at 3–15 passages. CAFs were cultured to approximately 70% confluence and then subjected to serum-free medium treatment for 48 h. The conditioned medium (CM) was then collected and filtered for subsequent investigation. WI-38 cells were transformed into CAFs by culturing in medium from various subtypes of breast cancer cells for 48 h, as described previously [23, 24]. Subsequently, CAF-CM from activated WI-38 cells was then used to stimulate breast cancer cells for an additional 48 h.

### Wound healing assay

TNBC cells were stained with CellTracker™ Green dye for 30 min at 37 ℃, digested, and co-cultured with CAFs at a ratio of 2:1 in 6-well plates. Once the cells reached approximately 90% confluence, the cell monolayers were scraped using a micropipette tip and treated with or without shikonin in serum-free medium. Alternatively, TNBC cells were seeded in 6-well plates, scraped, and treated with or without CAF-CM. The scratches in the cell monolayers were photographed under a microscope (DMI8; Leica, Wetzlar, Germany) at 0 and 48 h after treatment.

### Cell invasion assay

Transwell chambers, pre-coated with Matrigel (8-μm pore size) and placed in 24-well plates, were used to assess cell invasion. TNBC cells, pre-treated with or without shikonin for 6 h, were seeded in the upper compartment with serum-free media, and CAFs in serum-free medium were included in the lower compartment. Alternatively, TNBC cells were treated with CAF-CM with or without shikonin for 48 h, then suspended with serum-free media in the upper compartment, while media containing 10% FBS was added into the lower compartment. The Transwell systems were placed in a humidified incubator for 24 h (5% CO_2_, 37 ℃). The infiltrated cells were fixed with methanol, stained with hematoxylin/eosin, and photographed using an inverted microscope (DMI1; Leica) followed by quantification with ImageJ software (v.1.8.0).

### Detection of filamentous actin (F-actin)

TNBC cells and CAFs (total 9 × 10^3^ cells per well, at a ratio of 2:1) were seeded into 96-well black plates. The cells were fixed (4%, paraformaldehyde, 25 ℃, 10 min), permeabilized (0.2% Triton X-100, 4 ℃, 10 min), blocked (5%, goat serum, 25 ℃, 30 min), and then incubated with Alexa Fluor 488™ phalloidin (1:50, 37 ℃, 30 min) for F-actin staining. The cells were then incubated with anti-EpCAM antibody (1:500, 4 ℃, overnight) followed by Alexa Fluor 647 secondary antibody (1:250, 25 ℃, 1.5 h), and stained with 4’,6-diamidino-2-phenylindole (DAPI, 1 μg/mL, 25 ℃, 30 min) for nuclear staining. Fluorescence signals were detected using a high-content imaging system.

### 3D-culture assay

The 24-well plates were pre-coated with Matrigel (growth factor reduced, GFR, 300 μL/well). TNBC cells were collected and resuspended in media containing 2% GFR Matrigel, and then located in the Matrigel-coated plates. The cells were then treated with CAF-CM with or without shikonin, and the treatment medium was replaced every day for 3 days. The cells were photographed under a DMI1 microscope (Leica).

### Detection of anoikis

TNBC cells were inoculated in poly-2-hydroxyethyl methacrylate coated (anchorage-resistant) plates for 24 h, incubated with propidium iodide (1:100) and Annexin V-fluorescein isothiocyanate (1:100) reagents at 25℃ for 15 min, and then analyzed using flow cytometry (NovoCyte; Ex/Em = 488/630; 488/530 nm) with NovoExpress software (ACEA Biosciences). Alternatively, TNBC cells were incubated with calcein AM (1:5) and ethidium homodimer (EthD-1, 1:5) from a CytoSelect™ 96-Well Anoikis Assay kit for 30 min at 37 ℃. The fluorescence intensities of Calcein AM and EthD-1 were measured using a high-content analysis system at Ex/Em = 485/515 and 525/590 nm, respectively, with Harmony Software (v.4.9; PerkinElmer, Berlin, Germany).

### Detection of mitochondrial numbers and distribution

Mitochondrial numbers were assessed by staining the cells with MitoTracker Green FM (100 nM, 37 ℃, 30 min), followed by flow cytometry (NovoCyte; Ex/Em = 488/530 nm). Mitochondrial distribution was determined by staining the cells with Alexa Fluor 488™ phalloidin (1:50, 37 ℃, 30 min), MitoTracker Red (50 nM, 37 ℃, 30 min), and Hoechst 33342 (1:100, 25 ℃, 30 min) after fixation with 4% paraformaldehyde (25 ℃, 10 min). Images were captured using the high-content analysis system.

### Quantification of mitochondrial DNA (mtDNA) levels

This experiment was performed using a genomic DNA extraction kit, TB Green Premix, and Human Mitochondrial DNA Primer kit, as described previously [25].

### Transmission electron microscopy (TEM)

Cells were fixed in 2.5% glutaraldehyde (4 ℃, overnight), incubated in 1% osmium tetroxide (25 ℃, 2 h), dehydrated in a graded ethanol series, and then embedded with Epon 812. Ultrathin sections (70 nm) were obtained using an EM UC7 ultramicrotome (Leica) and counterstained with 2% uranyl acetate and lead citrate for 15 min. The sections were viewed using a compact digital transmission electron microscope at 80 kV (HT7800, Hitachi, Tokyo, Japan). Mitochondrial morphology was classified into three types based on the ratio of length to width using ImageJ software: ≤1.5: round; 1.5–3.0: intermediate; >3.0: elongated.

### Blue-native polyacrylamide gel electrophoresis (PAGE) analysis

Blue-native PAGE analysis was conducted as described previously [26]. Cells were collected and suspended in phosphate-buffered saline supplemented with digitalis glycoside (8 mg/mL), phenylmethanesulfonyl fluoride (PMSF, 1 mM), and protease inhibitor cocktail (PIC, 1%) at 4 ℃ for 15 min, followed by centrifugation (21,130 × *g*, 4 ℃, 20 min). The resulting pellets were resuspended in native PAGE sample buffer (containing 5% digitalis glycoside, 1 mM PMSF, and 1% PIC), vortexed (every 5 min for 30 min), and centrifuged (21,130 × *g*, 4 ℃, 30 min). The protein complexes were quantified using a BCA protein assay kit, separated using a native PAGE 4–16% gel, 10-well kit, and transferred to polyvinylidene fluoride membranes. After blocking with 5% nonfat-dried milk (4 ℃, 1 h), they were incubated with OXPHOS Rodent WB antibody cocktail (1:1000, 4 ℃, overnight) and secondary antibody. The results were imaged using an enhanced chemiluminescence kit in a ChemiDoc system (v.5.2 Image Lab Software; XRS^+^; Bio-Rad).

### Determination of mitochondrial reactive oxygen species (ROS) level

Mitochondrial ROS levels were assessed by staining cells with MitoSOX (4 μM) in serum-free medium (37 ℃, 30 min). The fluorescence intensities of MitoSOX (Ex/Em = 488/572 nm) were measured using flow cytometry with NovoExpress software (ACEA Biosciences).

### Detection of ATP levels

Cells were lysed in lysis buffer supplemented with 1 mM PMSF and 1% PIC. ATP levels were measured using an ATP determination kit and quantified with a microplate spectrophotometer (Varioskan, Thermo) at 560 nm. The ATP content was normalized to the cell protein concentration.

### Western blotting and immunoprecipitation assay

Total, nuclear, and mitochondrial proteins were extracted using radioimmunoprecipitation assay (RIPA) lysis buffer, a nuclear/cytoplasmic protein kit, and cell mitochondria isolation kit, respectively. The lysis buffer was supplemented with 1 mM PMSF and 1% PIC. Immunoprecipitation was performed using an immunoprecipitation kit. Western blotting was performed as described previously [25]. Immunoblots were detected using an enhanced chemiluminescence kit and a ChemiDoc system.

### Immunofluorescence staining

TNBC cells (6 × 10^3^ cells/well) were seeded into 96-well black plates, fixed (paraformaldehyde, 4%, 25 ℃, 10 min), permeabilized (Triton X-100, 0.2%, 4 ℃, 10 min), and blocked with 5% goat serum (25 ℃, 30 min). The cells were then incubated with anti-PGC-1α antibody (1:300, 4 ℃, overnight), followed by Alexa Fluor 488 secondary antibody (1:500, 25 ℃, 1.5 h), anti-ERRα antibody (1:200, 4 ℃, overnight), Alexa Fluor 647 secondary antibody (1:250, 25 ℃, 1.5 h), and DAPI (1 μg/mL, 25 ℃, 30 min). The results were detected using a high-content system.

### Dual-luciferase reporter assay

Cells were inoculated into 12-well plates and transfected with pRL Renilla luciferase control reporter vectors (pRL-TK) and ERRE-luciferase reporter plasmids (pGL3-3×ERRE) at a ratio of 1:10 using Lipofectamine 2000 reagent. After treatment, ERRE-luciferase activity was detected using a dual-luciferase reporter system and normalized to Renilla luciferase activity.

### Chromatin immunoprecipitation (ChIP) assay

ChIP assay was conducted with 2 × 10^7^ cells per group using a ChIP kit and anti-PGC-1α (5 μg) antibody according to the standard protocol. Quantification of PGC-1α ChIP enrichment was performed using a quantitative polymerase chain reaction (Q-PCR) detection system (Bio-Rad) and specific primers for the *ERRSA* promoter (Table S1). ChIP enrichment was calculated against the IgG group and subsequently normalized against the control group.

### Q-PCR

Q-PCR was performed using an RNA extraction kit, cDNA synthesis kit, and TB Green Premix in a CFX96 RT-PCR detection system (Bio-Rad), as described previously [25]. The primer sequences are listed in Table S1.

### Phosphorylation site and binding partners of PGC-1α

Cells were lysed using RIPA lysis buffer and immunoprecipitation-mass spectrometry (IP-MS) assays were performed. PGC-1α was immunoprecipitated from cellular extracts using an anti-PGC-1α antibody, and the immunoprecipitates were separated by sodium dodecyl sulfate-PAGE. The indicated protein was excised, digested with trypsin, fractionated, and then analyzed by liquid chromatography-tandem mass spectrometry (LC-MS/MS) using Easy-nLC 1000 and LTQ Orbitrap ETD (Thermo). Mass spectrometric data were identified using Proteome Discoverer software (Thermo; v1.4) and the NCBI database.

### Plasmid transfection

Lentiviruses harboring pGPU6/GFP/Neo-shRNA-LDHA vector (LDHA shRNA: 5′- CTCTGGCAAAGACTATAATGT-3′), pGPU6/GFP/Neo-shRNA-NC vector (5′- TTCTCCGAACGTGTCACGT-3′), LV2 (U6/Puro)-shRNA-PGC-1α vector (PGC-1α shRNA: 5′- GGTGCAGTGACCAATCAGAAA-3′), LV2-shRNA-NC vector (5′- TTCTCCGAACGTGTCACGT-3′), and pcDNA3.1 plasmids containing PGC-1α^WT^ or PGC-1α^A295^ were purchased form GenePharma (Shanghai, China). All transfection procedures were performed according to the manufacturer’s instructions. TNBC cells were transfected with shRNA-PGC-1α to knockdown PGC-1α and then transfected with pc DNA3.1 (+) (CMV/MCS/AmpR) plasmids to express PGC-1α^WT^- or PGC-1α^A295^. Cells with stable knockdown of PGC-1α and expression of PGC-1α^WT^ or PGC-1α^A295^ were selected by incubation with 0.2 μg/mL puromycin and 100 μg/mL ampicillin for 21 days.

### Animals and treatment

Female NOD/SCID mice (6–8 weeks old, 18–22 g, Charles River, Beijing, China) were raised in 12:12 h light/dark-cycle standard conditions and assigned randomly to different groups. MDA-MB-231 cells (MDA-MB-231/PGC-1α^WT^, MDA-MB-231/PGC-1α^A295^, 1 × 10^6^ cells/mouse) and CAF cells (0.5 × 10^6^ cells/mouse) were co-injected into the left fourth inguinal mammary fat pads (*n* = 10/group). After 4 weeks, tumors were removed by mammary gland lumpectomy. The following day, the mice were treated intragastrically with shikonin (10 mg/kg/2 days) or vehicle (olive oil) for 30 days. After the experiment, the mice were sacrificed under anesthesia using CO_2_ inhalation.

Tissues (5-μm sections) were stained with hematoxylin/eosin. TOM20 was detected in lung tissues by immunofluorescence using a fluorescence staining kit, anti-TOM20 antibody (1:200), and DAPI (1 μg/mL). Immunohistochemistry staining of lung tissues was conducted using a Biotin-Streptavidin HRP Detection kit with anti-PGC-1α (1:200), anti-ERRα (1:200), and anti-COXIV (1:200) antibodies. Tissue images were photographed using a DMI8 microscope and analyzed with ImageJ software. The investigators were blinded to the assignments and outcomes.

### Multiplex immunofluorescence staining

We carried out multiplex immunofluorescence staining of breast cancer (*n* = 230) and TNBC (*n* = 38) [27] tissue microarrays from patients with primary cancer (Shanghai Outdo Biotech, Shanghai, China). The clinicopathological parameters of these specimens are listed in Supplementary Tables S2 and S3. PGC-1α and ERRα expression in tumor tissues were evaluated using a PANO 7-plex immunohistochemistry kit, anti-PGC-1α, anti-ERRα, and anti-pan-cytokeratin (Pan-CK) antibodies, and DAPI. Fluorescence signals were captured using a Mantra System (PerkinElmer). PGC-1α and ERRα expression levels in breast cancer tissues were calculated by mean cell intensity, and PGC-1α expression in TNBC tissues was calculated by H-score using inForm image software. The pathologists who collated and analyzed the clinical parameters were blinded to the study results. The results were analyzed and represented using R script (v.4.0.2) and ggplot2 package.

### Kaplan–Meier plot analysis

The correlations between *PPARGC1A*, *ESRRA*, and *PPARGC1A*/*ESSRA* mRNA levels and RFS in patients with different breast cancer subtypes were analyzed using the Kaplan–Meier plotter database (http://kmplot.com, ER-positive/HER2-negative patients: *n* = 2301; HER-2-positive/ER-negative patients: *n* = 323; TNBC patients: *n* = 534) [28]. *PPARGC1A* and *ESRRA* mRNA levels were analyzed using the autoselect best cutoff mode, while *PPARGC1A/ESSRA* mRNA levels were analyzed using the mean expression levels of the selected genes. Statistical significance was calculated by log-rank test and indicated as *p* < 0.05. The correlations of *PPARGC1A* mRNA levels and mRNA levels of ERRα-targeted mitochondrial genes were evaluated using *R*- and *p*-values, calculated by Pearson’s correlation analysis. The results were represented using R script (v.4.0.2) and the ggplot2 package.

### Statistical analysis

The results are presented as the mean ± standard deviation. Differences among multiple groups were analyzed by one way-ANOVA and Bonferroni’s *post hoc* test, while differences between two groups were analyzed by Student’s *t*-test. Differences in Kaplan–Meier survival curves were evaluated using the log-rank test. A *p* value < 0.05 was considered statistically significant in all tests conducted in this study.

## Results

### Shikonin suppresses CAF-enhanced metastatic ability in TNBC cells

**Fig. 1:**
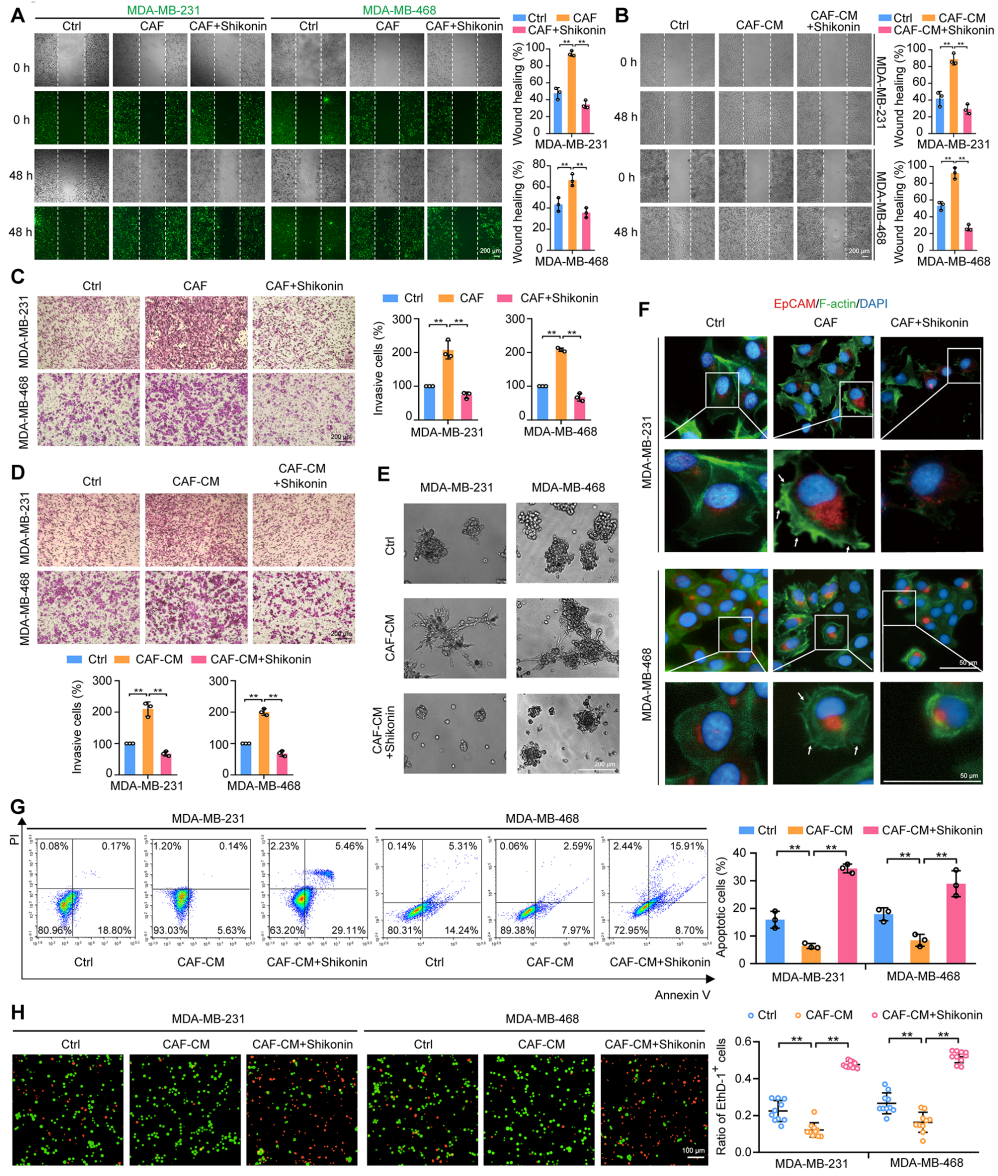
Shikonin inhibits CAF-stimulated metastatic potential of TNBC cells. (**A**) Cell migration ability was measured by the wound-healing assay. TNBC cells (stained with CellTracker™ Green, green fluorescence) were co-cultured with CAFs at a ratio of 2:1 and treated with or without shikonin (2 μM) for 48 h. Images were captured at 0 and 48 h post-wounding (magnification, ×50; scale bars, 200 μm, *n* = 3). (**B**) Cell migration was measured by wound-healing assay. TNBC cells were treated with CAF-CM in the presence or absence of shikonin (2 μM) for 48 h. Images were captured at 0 and 48 h post-wounding (magnification, ×50; scale bars, 200 μm, *n* = 3). (**C**) Cell invasion was measured by Transwell assay. TNBC cells were pretreated with shikonin (2 μM) for 6 h and then inoculated in the upper compartment, and CAFs were inoculated in the lower compartment. TNBC cells and CAFs were cocultured for 24 h and images were captured (magnification, ×100; scale bars, 200 μm, *n* = 3). (**D**) Cell invasion was measured by Transwell assay. TNBC cells were treated with CAF-CM in the presence or absence of shikonin (2 μM) for 48 h. Images were captured (magnification, ×100; scale bars, 200 μm, *n* = 3). (**E**) TNBC cells grown in Matrigel. TNBC cells were treated with CAF-CM in the presence or absence of shikonin (2 μM) for 3 days (magnification, ×50; scale bars, 200 μm). (**F**) Immunofluorescence staining of TNBC cells labeled with anti-EpCAM antibody (red fluorescence), F-actin (green fluorescence), and DAPI (blue fluorescence). TNBC cells were co-cultured with CAFs at a ratio of 2:1 and treated with or without shikonin (2 μM) for 48 h. Arrows indicate filopodia and lamellipodia (magnification, ×400; scale bars, 50 μm). Cells were treated with or without shikonin (2 μM) in the presence or absence of CAF-CM for 48 h. (**G, H**) Anoikis of TNBC cells was detected by Annexin V-fluorescein isothiocyanate/propidium iodide (PI) double staining (**G**; *n* = 3) or calcein AM/ethidium homodimer (EthD-1) staining (**H**; magnification, ×200; scale bars, 100 μm; *n* = 10). Data are presented as mean ± SD. ^**^p < 0.01

The inhibitory effects of shikonin on CAF-promoted metastasis in TNBC cells were assessed using a coculture of TNBC cells and CAFs, and stimulated by CAF-CM. TNBC cells (MDA-MB-231 and MDA-MB-468 cells) were labeled with CellTracker™ Green. Cell migration was assessed and the results showed that TNBC cells cocultured with CAFs (at a ratio of 2:1) and stimulated with CAF-CM displayed enhanced migration ability (Fig. 1A, B). Cell invasion assay using a Matrigel-coated Transwell system indicated that coculture with CAFs and CAF-CM promoted invasion of TNBC cells (Fig. 1C, D). CAF-enhanced migration and invasion were notably diminished by shikonin (Fig. 1A–D). Moreover, TNBC cells treated with CAF-CM exhibited a more spherical and immotile morphology and formed more protrusions in 3D Matrigel cultures. These protrusions interacted with each other, attracting more clusters and thus resulting in larger cell clusters, which were also reversed by shikonin treatment (Fig. 1E). TNBC cells stimulated by CAFs exhibited cytoskeletal remodeling, marked by the formation of filopodia and lamellipodia. In contrast, shikonin inhibited these CAF-induced morphological changes in TNBC cells (Fig. 1F). In addition to increased migration and invasion, we also examined anoikis resistance, as an important characteristic of cancer cells during metastasis [29]. Stimulation with CAF-CM reduced anoikis while shikonin increased anoikis in TNBC cells (Fig. 1G, H). Collectively, these data indicate that shikonin suppressed CAF-enhanced metastatic capabilities in TNBC cells.

### Shikonin attenuates CAF-stimulated mitochondrial biogenesis and energetics

**Fig. 2:**
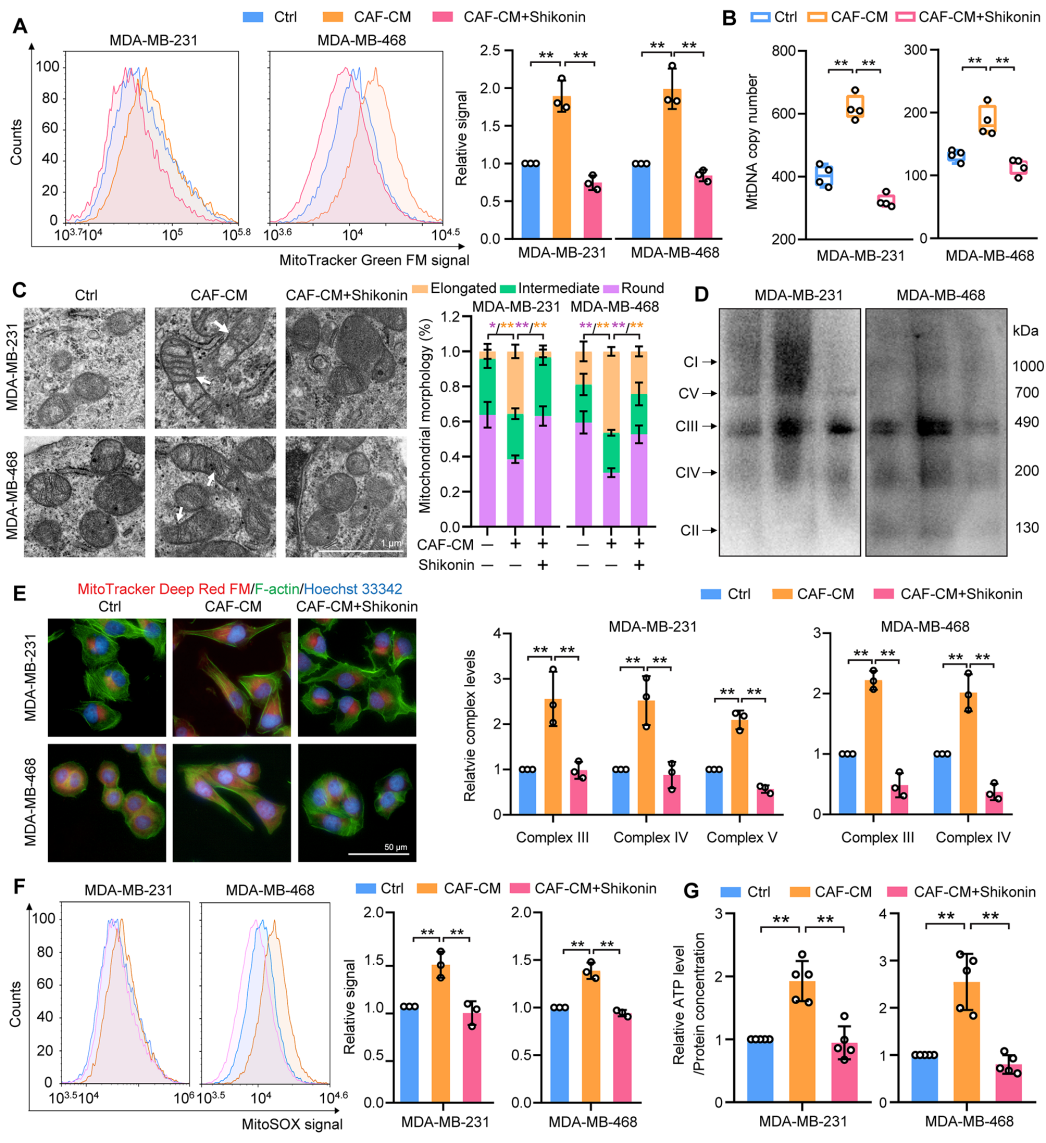
Shikonin modulates mitochondrial biogenesis in TNBC cells in response to CAF stimulation. Cells were treated with CAF-CM in the presence or absence of shikonin (2 μM) for 48 h. (**A**) Mitochondria were visualized using MitoTracker Green staining and analyzed by flow cytometry (*n* = 3). (**B**) Evaluation of mtDNA levels relative to ND1 and ND5, compared with SLCO2B1 and SERPINA1 as controls (*n* = 4). (**C**) Transmission electron microscopy depicting mitochondrial morphology in TNBC cells (magnification ×15 000; scale bar, 1 μm). Quantification of mitochondria with different morphologies is shown (*n* = 5). (**D**) Detection of mitochondrial complexes using native polyacrylamide gel electrophoresis and a mitochondrial antibody cocktail. The quantification of the data was represented by the fold-changes compared to the control groups (*n* = 3). (**E**) Visualization of mitochondrial distribution using MitoTracker Deep Red FM (red fluorescence, mitochondria), Alexa Fluor 488™ phalloidin (green fluorescence, F-actin), and Hoechst33342 (blue fluorescence, nucleus) staining (magnification, ×400; scale bars, 50 μm). (**F**) Assessment of mitochondrial reactive oxygen species (ROS) levels using MitoSOX labeling flow cytometry (*n* = 3). (**G**) ATP content in cellular extracts was calculated using an ATP detection kit. Intracellular ATP levels were normalized to the protein concentration (*n* = 5). Data are presented as mean ± SD. ^*^p < 0.05, ^**^p < 0.01

TNBC cells demonstrate a predilection for mitochondrial metabolism during metastasis [5, 10, 30]. We therefore explored if shikonin influenced mitochondrial biogenesis in CAF-stimulated TNBC cells by examining the number of mitochondria and mtDNA content. CAF-CM stimulation increased the mitochondria count and mtDNA content in TNBC cells, which were inhibited by shikonin treatment (Fig. 2A, B). Regarding mitochondrial morphology, which is indicative of metabolic pathway alterations [31], CAF-stimulated TNBC cells displayed elongated and larger mitochondria with numerous cristae, signifying heightened electron transport chain performance and energy production [32], which were countered by shikonin treatment (Fig. 2C). In addition, CAF-CM treatment upregulated mitochondrial respiratory complexes III, IV, and V in MDA-MB-231 cells and complexes III and IV in MDA-MB-468 cells, and these were reduced by shikonin (Fig. 2D). CAF-CM also prompted the localization of more mitochondria to the plasma membrane in TNBC cells to support ATP production necessary for actin filament polymerization and lamellipodia formation during cell migration [33], while shikonin treatment resulted in a predominant mitochondrial distribution near the nucleus (Fig. 2E). Activation of mitochondrial respiration typically increases mitochondrial ROS formation and cellular ATP production [34]. Consistently, elevated mitochondrial ROS and ATP levels were observed in CAF-CM-stimulated TNBC cells, which were suppressed by shikonin (Fig. 2F, G). Lactate, a crucial metabolite produced by CAFs [12], is synthesized from pyruvate by lactate dehydrogenase A (LDHA), a key component of the glycolytic pathway that also generates ATP [35]. To investigate the role of lactate from CAFs in mitochondrial biogenesis, we employed shRNA to knock down LDHA and subsequently reduce L-Lactate level in CAF-CM (Fig. S2A and 2B), and investigated the role of lactate from CAFs in mitochondria biogenesis. LDHA knockdown significantly limited the effect of CAF-CM on mitochondrial biogenesis (Fig. S2C-F), indicating that lactate was required for CAF-induced mitochondrial biogenesis in TNBC cells. Furthermore, we found that shikonin reduced CAF-CM-induced mitochondrial biogenesis from shNC-transfected CAFs, but not shLDHA-transfected CAFs (Fig. S2C-F). Shikonin also diminished the lactate-upregulated mitochondria contents, mtDNA levels, mitochondrial ROS, and ATP production in TNBC cells (Fig. S2G-J). However, the impact of shikonin alone on mitochondrial biogenesis in TNBC cells were minimal (Fig. S3A–D). These data indicate that the CAF-induced mitochondrial biogenesis and energetics can be suppressed by shikonin in TNBC cells.

### Shikonin represses CAF-induced PGC-1α expression and activation in TNBC cells

**Fig. 3:**
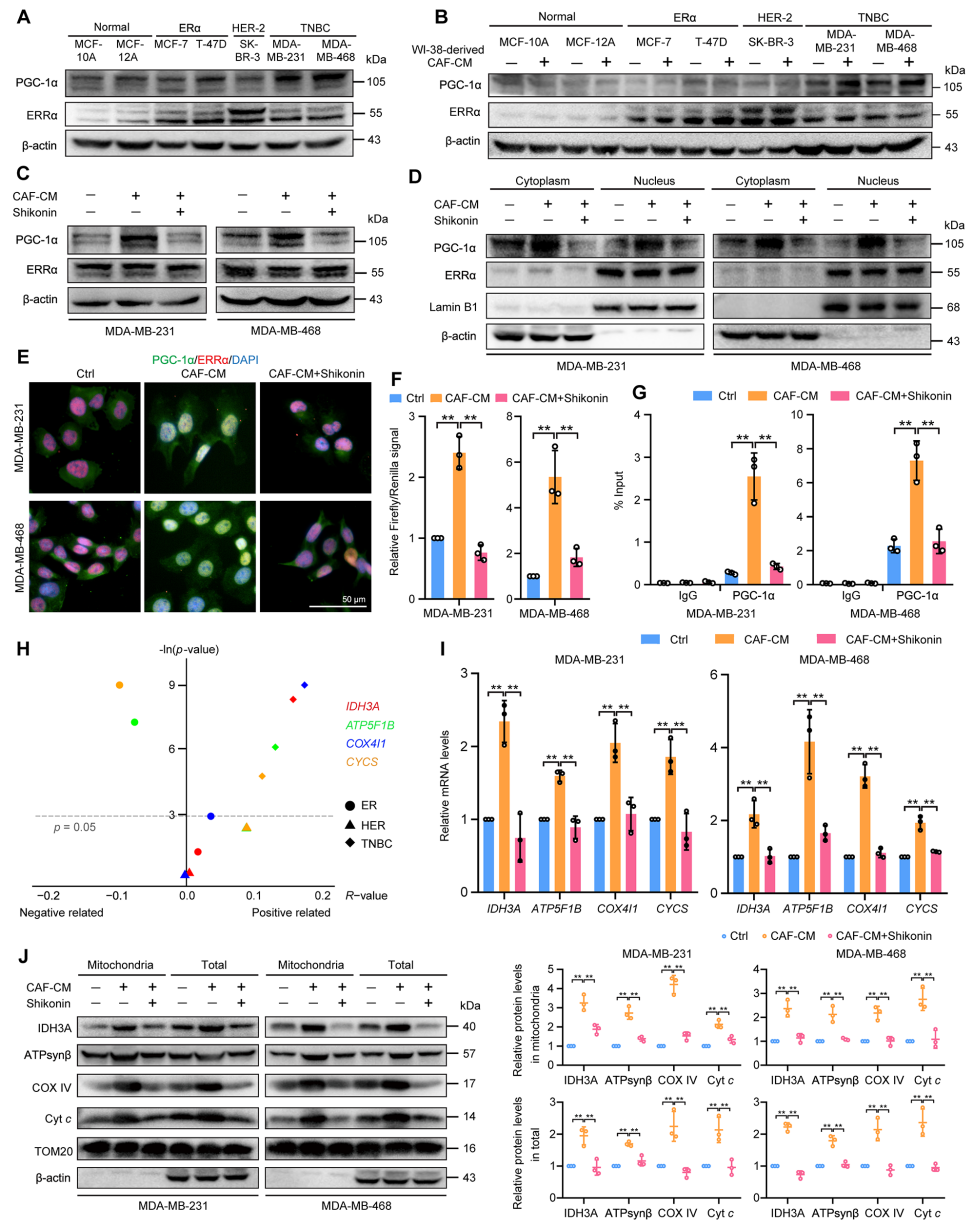
Shikonin modulates the CAF-induced PGC-1α/ERRα axis in TNBC cells. The culture medium from different subtypes breast cancer cells was used to activate WI-38 cells for 48 h, stimulating them to transform into CAFs. Then CAF-CM from activated WI-38 cells was used to induce breast cancer cells for 48 h in both (A) and (B). (**A**) Expression of PGC-1α and ERRα in different breast cancer cell types. (**B**) Expression of PGC-1α and ERRα in different breast cancer cell types after WI-38 cell transformed CAF-stimulation. TNBC cells were treated with CAF-CM in the presence or absence of shikonin (2 μM) for 48 h: (C)-(F). (**C**) PGC-1α and ERRα expression in TNBC cells. (**D**) Cytoplasmic and nuclear distributions of PGC-1α and ERRα in TNBC cells. Lamin B1 and β-actin were used as endogenous references for nuclear fractions and cytosolic lysates, respectively. (**E**) Cellular localization of PGC-1α and ERRα in TNBC cells detected by immunofluorescent staining (magnification, ×400; scale bars, 50 μm). (**F**) Transcriptional activity of ERRα in TNBC cells. TNBC cells were transfected with the pRL Renilla luciferase vector (pRL-TK) and the ERRE-luciferase reporter plasmid (pGL3-3×ERRE) at 1:10 (*n* = 3). (**G**) ERRα target promoter occupancy in TNBC cells evaluated using chromatin immunoprecipitation-RT-PCR with a PGC-1α antibody (*n* = 3). (**H**) Correlation between *PPARGC1A* and ERRα-targeted genes (*IDH3A*, *ATP5F1B*, *COX4I1*, *CYCS*) in breast cancer specimens from different breast cancer subtypes. The correlations were evaluated using *R*-value and *p*-value which were calculated by Pearson correlation analysis. The results were represented using R script (v.4.0.2) and ggplot2 package. The *p*-value was transformed to –ln (*p*-value). *PPARGC1A*: 219195_at; *IDH3A*: 202070_s_at, *ATP5F1B*: 211755_s_at; *COX4I1*: 213758_at; *CYCS*: 208905_at. (**I**) mRNA levels of *IDH3A*, *ATP5F1B*, *COX4I1*, and *CYCS* were detected using RT-PCR. Results were normalized to *ACTB* mRNA levels and reported as fold-change compared with control cells (*n* = 3). (**J**) Protein levels of IDH3A, ATPsynβ, COX IV, and Cyt *c* in mitochondria and total cells detected by western blotting. TOM20 and β-actin were used as endogenous references for mitochondrial fractions and total lysates, respectively. Data are presented as mean ± SD (*n* = 3). ^**^p < 0.01

It has been revealed that PGC-1α/ERRα axis regulates mitochondrial biogenesis in cancer cells [36]. We therefore investigated the regulation of the PGC-1α/ERRα axis in CAF-induced TNBC cells and assessed the impact of shikonin on this pathway. PGC-1α expression was more elevated in TNBC cell lines compared with other breast cancer subtypes and normal breast epithelial cells (Fig. 3A). ERRα expression was higher in ER-positive and HER-2-positive breast cancer cell lines (Fig. 3A). We utilized various types of breast cancer cells to stimulate WI-38 cells to undergo their transformation into CAFs (Fig. S4A). PGC-1α levels were significantly increased in CAF-stimulated TNBC cells compared with CAF-stimulated ER-positive and HER-2-positive cells (Fig. 3B), but ERRα expression did not exhibit a notable change in these cells following CAF stimulation (Fig. 3B). PGC-1α was suggested to be specifically upregulated in TNBC cells in response to CAF-stimulation. Consistently, we observed that PGC-1α levels increased in CAF-induced TNBC cells, whereas ERRα levels did not change (Fig. 3C). Additionally, the levels of PGC-1α in TNBC cells were also upregulated by CAF-derived lactate (Fig. S4B), and the migration, invasion and protrusion formation promoted by CAFs in TNBC cells were reversed upon PGC-1α knockdown (Fig. S4C-F). Western blot and immunofluorescence staining revealed that ERRα was predominantly localized in the nucleus (indicated by purple fluorescence) and remained unchanged following CAF-CM treatment (Fig. 3D, E). In contrast, PGC-1α, typically localized in the cytoplasm, and the nuclear localization of both PGC-1α and ERRα increased (shown by light-yellow fluorescence) after exposure to CAF-CM (Fig. 3D, E). Shikonin did not alter ERRα expression or its subcellular distribution; however, it significantly reduced CAF-CM-induced increase in PGC-1α expression and its nuclear localization in TNBC cells (Fig. 3C–E). Luciferase reporter results showed that CAF-CM enhanced the ERRα transcriptional activity, which was diminished by cotreatment with shikonin (Fig. 3F). Furthermore, ChIP assay confirmed that shikonin counteracted the CAF-induced promotion of PGC-1α binding to the ERRα promoter, suggesting shikonin inhibits the transcriptional activity of the PGC-1α/ERRα complex induced by CAFs (Fig. 3G).

To further evaluate the inhibitory effects of shikonin on PGC-1α/ERRα axis-targeted mitochondrial gene, we firstly established the positive correlations between *PPARGC1A* mRNA levels and ERRα-targeted mitochondrial genes mRNA levels, including *IDH3A* (isocitrate dehydrogenase 3 (NAD+) α, IDH3A), *ATP5F1B* (ATP synthase β, ATPsynβ), *COX4I1* (cytochrome c oxidase subunit 4I1, COX IV), and *CYCS* (cytochrome c, Cyt *c*), which are involved in the TCA cycle and respiratory chain components, respectively, in TNBC breast cancer tissues rather than ER-positive or HER-2-positive breast cancer tissues, using Kaplan–Meier plot database (Fig. 3H). Data showed that CAF-CM treatment led to elevated levels of these mitochondrial genes (Fig. 3I). Correspondingly, the total and mitochondrial protein levels of IDH3A, ATPsynβ, COX IV, and Cyt *c* were also increased following CAF-CM treatment (Fig. 3J). These results suggest that CAFs upregulate and activate PGC-1α, which in turn boosts ERRα transcriptional activity in TNBC cells. Conversely, shikonin suppresses PGC-1α expression and function, leading to repression of the transcriptional activities of the PGC-1α/ERRα axis.

### Shikonin promotes PGC-1α phosphorylation facilitating its ubiquitination-mediated degradation

**Fig. 4:**
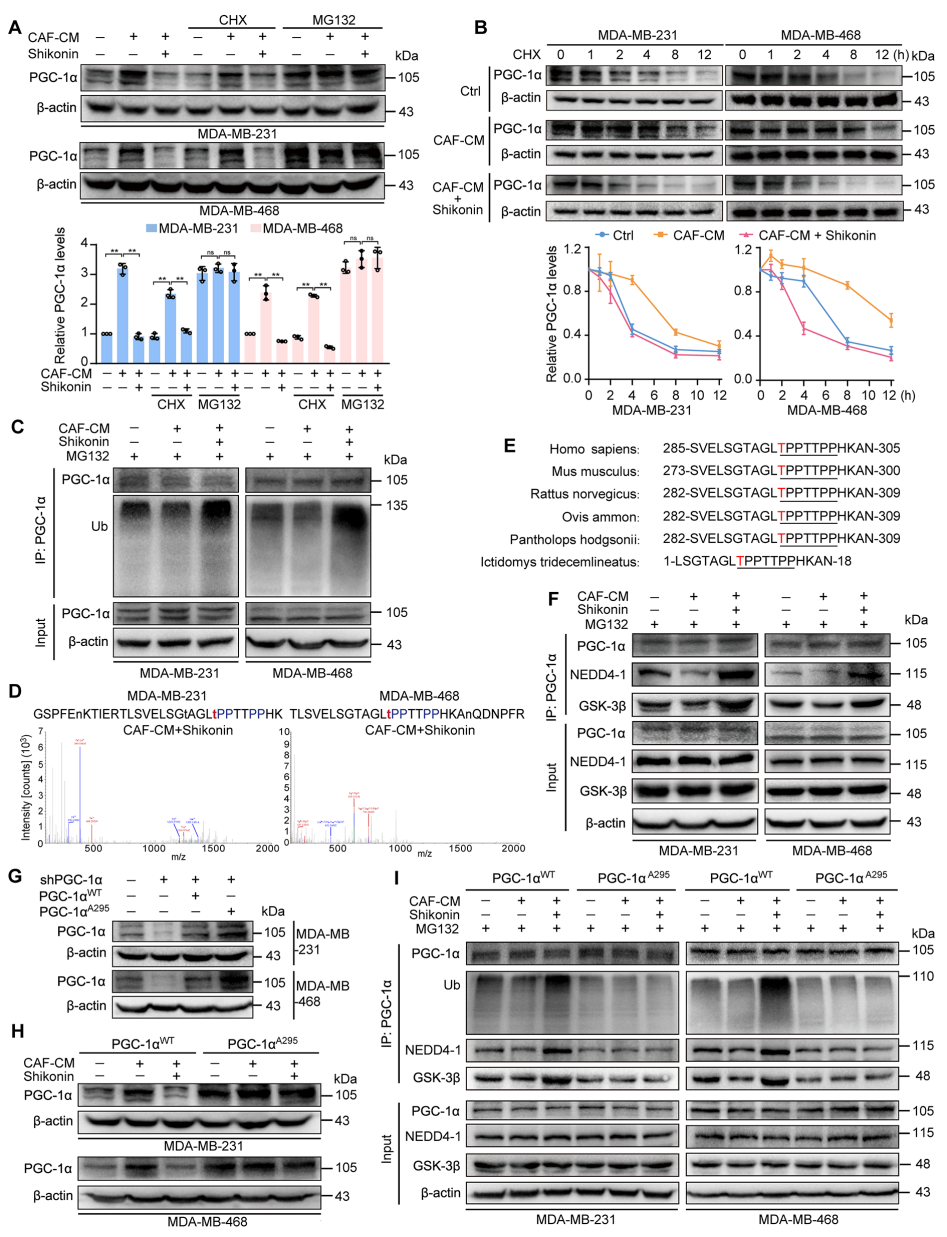
Shikonin enhances phosphorylation of PGC-1α, facilitating ubiquitination-mediated degradation. TNBC cells were treated with CAF-CM in the presence or absence of shikonin (2 μM) for 48 h. (**A**) PGC-1α expression in TNBC cells under indicated treatment. Cells were pretreated with cycloheximide (6 μM) for 12 h or MG132 (10 μM) for 6 h, and treated with or without CAF-CM ± shikonin for 48 h (*n* = 3). (**B**) Shikonin shortened the half-life of PGC-1α protein in CAF-stimulated TNBC cells. Cells were treated with cycloheximide (6 μM) with or without CAF-CM ± shikonin for the indicated time period and then harvested for western blotting (*n* = 3). The results were presented as fold change compared with the respective treated group at 0 h. (**C**) Shikonin promoted polyubiquitination of PGC-1α protein in TNBC cells. Cells were treated with MG132 (10 μM) for 12 h and harvested for western blotting. (**D**) Identification of phosphorylation sites of PGC-1α in TNBC cells. Immunoprecipitation experiments were conducted using an anti-PGC-1α antibody, and the phosphorylation sites of PGC-1α were identified by LC-MS/MS. (**E**) Alignment of PGC-1α protein sequence spanning T295 in different species. (**F**) Shikonin increased the interaction of PGC-1α with NEDD4-1 and GSK-3β in TNBC cells. PGC-1α was immunoprecipitated from whole-cell lysates using an anti-PGC-1α antibody, and the lysates and immunoprecipitate were blotted with PGC-1α, and NEDD4-1 and GSK-3β antibodies. (**G**) PGC-1α expression in TNBC cells transfected with shNC or shPGC-1α followed by transfection with PGC-1α^WT^ and PGC-1α^A295^ plasmids, respectively. (**H**) Mutation of PGC-1α at Thr295 reversed the inhibitory effects of shikonin on PGC-1α expression in TNBC cells. (**I**) Mutation of PGC-1α at Thr295 reversed the shikonin-promoted PGC-1α polyubiquitination and its interaction with NEDD4-1 and GSK-3β in TNBC cells. Data are presented as mean ± SD, **p < 0.01, ns: not significant

To investigate the mechanism by which shikonin suppresses PGC-1α expression, a protein synthesis inhibitor cycloheximide (CHX) and a proteasome inhibitor MG132 were firstly employed. Data revealed that MG132, but not CHX, reversed the inhibitory effects of shikonin on CAF-CM-induced PGC-1α expression (Fig. 4A). However, *PPARGC1A* mRNA levels remained unchanged with or without shikonin treatment in the presence of CAF-CM (Fig. S5). Shikonin shortened the extended half-life of PGC-1α caused by CAF-CM in CHX-treated TNBC cells (Fig. 4B). Additionally, CAF-CM decreased, while shikonin enhanced, the ubiquitination of PGC-1α in both TNBC cell lines (Fig. 4C).

Since ubiquitin-mediated proteolytic degradation of PGC-1α is phosphorylation-dependent [37], we immunoprecipitated TNBC cell lysates using an anti-PGC-1α antibody and subjected them to LC-MS/MS. We highlighted the most enriched phosphorylation site in each group (Fig. S6). Shikonin increased PGC-1α phosphorylation on threonine 295 (Thr295) in CAF-CM-treated TNBC cells (Fig. 4D). Continuing with IP-MS, we screened out 205 proteins potentially associated with PGC-1α in the pulldown samples from MDA-MB-231 and MDA-MB-468 cells treated with shikonin under CAF stimulation (Fig. S7A, Table S4). Among these proteins, glycogen synthase kinase 3β (GSK-3β), known for phosphorylating PGC-1α on Thr295 [38], and neural precursor cell expressed developmentally downregulated 4e1 (NEDD4-1, or NEDD4), an E3 ubiquitin ligase with a poly-proline rich (PPR) binding domain recognizing PPR motifs in substrates [39], may mediate shikonin-induced ubiquitin-proteasome degradation of PGC-1α that contains a PPR motif (Fig. S7A and Fig. 4D), and which is conserved among species (Fig. 4E).

To validate this hypothesis, we confirmed that shikonin increased the interaction between PGC-1α and GSK-3β or NEDD4-1 in TNBC cells, under CAF-CM stimulation (Fig. 4F). Additionally, GSK-3β and NEDD4-1 were upregulated in TNBC cells following shikonin treatment, with or without CAF-CM incubation (Fig. S7B). To confirm the effects of shikonin on PGC-1α phosphorylation at Thr295 which is responsible for its degradation, we generated an unphosphorylated mutant PGC-1α, replacing Thr295 with alanine (Ala295). Cells were transfected with shPGC-1α to knockdown endogenous PGC-1α, followed by transfection with plasmid expressing either wild-type (PGC-1α^WT^) or mutant PGC-1α (PGC-1α^A295^) (Fig. 4G). Shikonin reduced CAF-CM-enhanced PGC-1α levels in PGC-1α^WT^ cells, but had no effect in PGC-1α^A295^ cells (Fig. 4H). Similarly, the half-life of PGC-1α^WT^ was shortened by shikonin; however, the half-life of PGC-1α^A295^ were unaffected by CAF-CM with or without shikonin treatment (Fig. S7C). Furthermore, the Ala295 mutation reduced the interaction of PGC-1α with GSK-3β and NEDD4-1 in TNBC cells (Fig. 4I). Shikonin thus promoted the association of PGC-1α with GSK-3β and NEDD4-1, as well as PGC-1α ubiquitination in PGC-1α^WT^- but not in PGC-1α^A295^-expressing cells (Fig. 4I, Fig. S7D). These results indicate that shikonin promotes PGC-1α phosphorylation at Thr295 by GSK-3β, which strengthens its interaction with NEDD4-1, leading to increased PGC-1α ubiquitination and degradation.

### Shikonin inhibits mitochondrial biogenesis and metastasis via phosphorylation-dependent PGC-1α degradation in TNBC cells

**Fig. 5:**
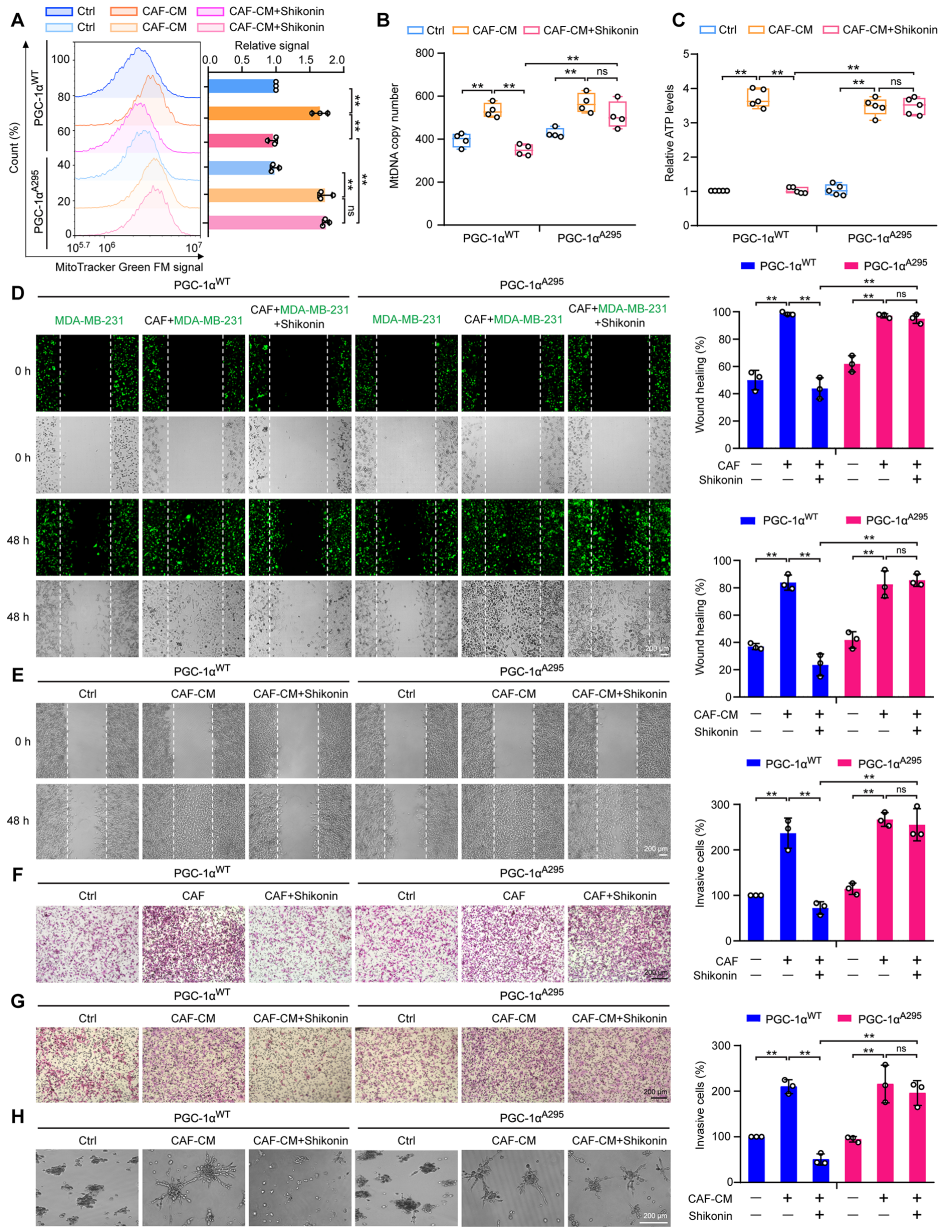
Mutation of PGC-1α at Thr295 reverses the inhibitory effects of shikonin on CAF-stimulated metastasis and mitochondrial biogenesis in TNBC cells. (**A**) Detection of mitochondria using MitoTracker Green FM staining and flow cytometry (*n* = 3). (**B**) Evaluation of mtDNA levels relative to ND1 and ND5, compared with SLCO2B1 and SERPINA1 as controls (*n* = 4). (**C**) Calculation of ATP content in cellular extracts using an ATP detection kit. Intracellular ATP levels were normalized to the protein concentration (*n* = 5). (**D**) Cell migration of MDA-MB-231 cells co-cultivated with CAFs at a ratio of 2:1 with or without shikonin (2 μM) treatment for 48 h. Cells were stained with CellTracker™ Green (green fluorescence). Images were captured at 0 and 48 h following wounding (magnification, ×50; scale bars, 200 μm, *n* = 3). (**E**) Migration of MDA-MB-231 cells detected using wound healing assays with CAF-CM in the presence or absence of shikonin (2 μM). Images were captured at 0 and 48 h following wounding (magnification, ×50; scale bars, 200 μm, *n* = 3). (**F**) Cell invasion ability measured using Transwell assays in MDA-MB-231 cells cocultured with CAFs after pretreatment with or without shikonin (2 μM) treatment for 6 h. (magnification, ×100; scale bars, 200 μm, *n* = 3). (**G**) Cell invasion of MDA-MB-231 cells measured using Transwell assays after CAF-CM stimulation with or without shikonin (2 μM) for 48 h (magnification, ×100; scale bars, 200 μm, *n* = 3). (**H**) 3D-culture of MDA-MB-231 cells treated with CAF-CM in the presence or absence of shikonin (2 μM) for 3 d (magnification, ×50; scale bars, 200 μm). Data are presented as mean ± SD. ^**^p < 0.01, ns: not significant

To elucidate the role of phosphorylation-mediated degradation of PGC-1α in the inhibitory activity of shikonin, we reintroduced PGC-1α^WT^ or PGC-1α^A295^ plasmids into PGC-1α-deficient MDA-MB-231 cells. After priming with CAF-CM, shikonin treatment resulted in decreased mitochondrial numbers, mtDNA levels, and ATP production in PGC-1α^WT^ cells. However, it failed to reduce mitochondrial biogenesis in PGC-1α^A295^ cells (Fig. 5A–C). Moreover, CAF-coculture- or CAF-CM-enhanced migration and invasion were reduced by shikonin in PGC-1α^WT^ cells, but not in PGC-1α^A295^ cells (Fig. 5D–G). Moreover, shikonin also impaired the formation of invasive outward projections in PGC-1α^WT^ cells under a 3D-culture system stimulated with CAF-CM (Fig. 5H). Thus, mutating PGC-1α at the Thr295 phosphorylation site abolished the effects of shikonin.

**Fig. 6:**
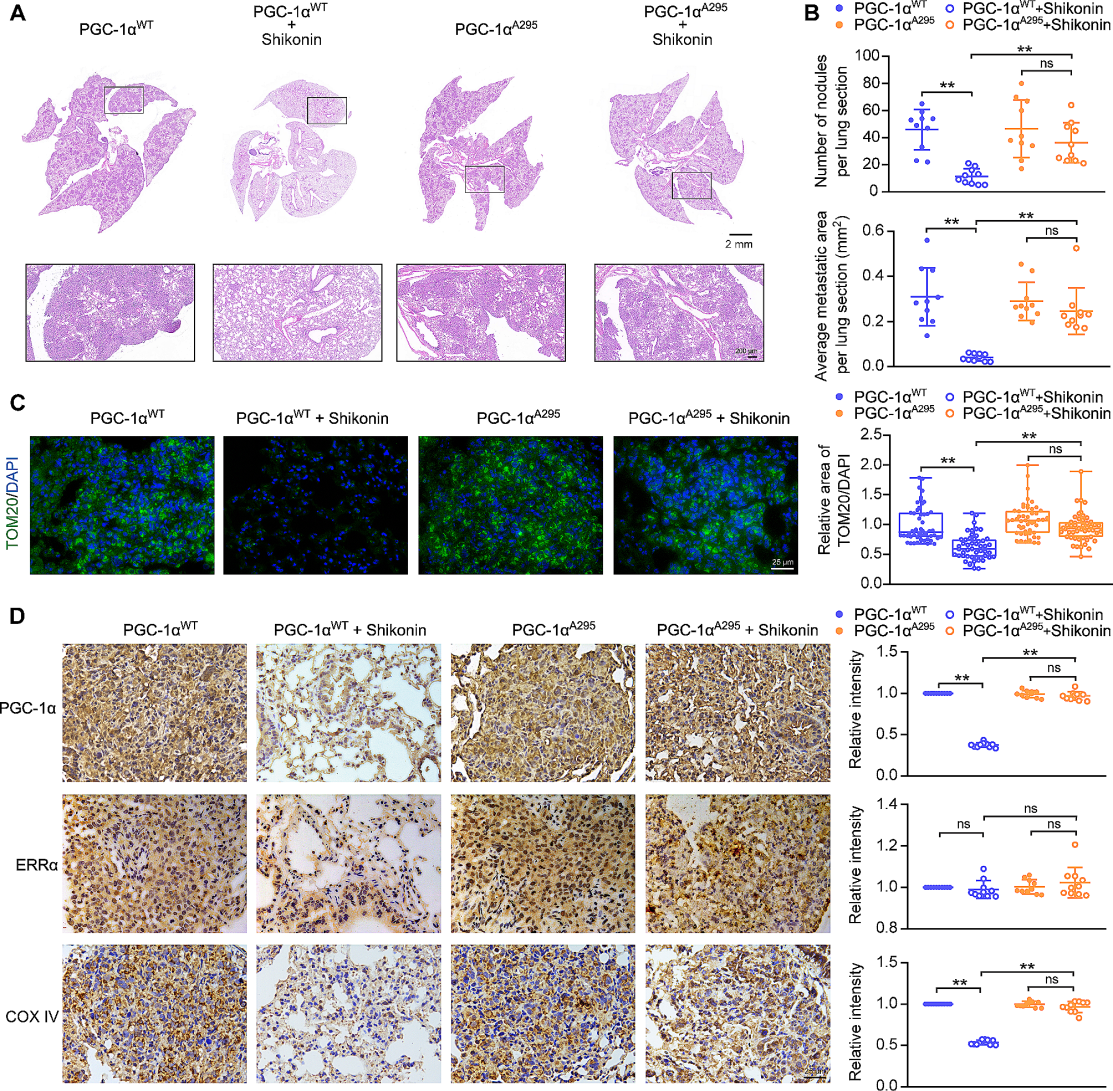
Mutation of PGC-1α at Thr295 reverses the inhibitory effects of shikonin on CAF-stimulated metastasis in TNBC cells in vivo. (**A**) HE staining of lung section (scale bars: 2 mm; 200 μm). (**B**) Quantification of lung metastasis. Number of nodules and average area of metastatic foci per lung section (*n* = 10). (**C**) Representative images of lung sections with immunofluorescent staining of TOM20 expression (magnification, ×400; scale bars, 25 μm). Five tumor nodules were selected in a lung section, and lung sections from ten different lung sections were used (*n* = 50). The ratio of mitochondrial area to nuclear area was calculated using ImageJ software. (**D**) Representative images of lung sections immunostained for PGC-1α, ERRα, and COX IV (magnification, ×400; scale bars, 25 μm). The intensities of PGC-1α, ERRα, and COX IV per lung section were calculated using ImageJ software from ten different lung sections (*n* = 10). Data are presented as mean ± SD. ^**^p < 0.01, ns: not significant

To further explore the role of PGC-1α phosphorylation at Thr295 in the response of TNBC cells to shikonin in vivo, we implanted PGC-1α^WT^ or PGC-1α^A295^ cells along with CAFs (at a 2:1 ratio) into the mammary fat pads of NOD/SCID female mice. After removing the primary tumors, the mice were treated with or without shikonin for 30 days. Both cell types formed noticeable lung metastases under CAF stimulation (Fig. 6A). Shikonin treatment significantly reduced lung metastases in mice implanted with PGC-1α^WT^-expressing MDA-MB-231 cells, as evidenced by a marked decrease in metastatic lesions and lung metastatic foci area (Fig. 6B). Notably, shikonin did not cause significant loss of body weight or damage to the hematological system and visceral organs (Fig. S8A, B, Table S5, S6). However, the inhibitory effects of shikonin on lung metastasis were not observed in mice implanted with PGC-1α^A295^ MDA-MB-231 cells (Fig. 6A, B).

With the tissue sample, mitochondria were detected by immunofluorescent staining of TOM20. Shikonin treatment reduced mitochondria levels in lung metastases in PGC-1α^WT^, but not in those expressing PGC-1α^A295^ (Fig. 6C). Consistent with the in vitro findings, ERRα expression in lung tissues in mice transplanted with either PGC-1α^WT^- or PGC-1α^A295^ MDA-MB-231 cells remained unaffected after shikonin treatment (Fig. 6D). However, shikonin inhibited PGC-1α and COX IV expression in pulmonary metastases of PGC-1α^WT^ cells but not in those expressing PGC-1α^A295^ (Fig. 6D). These results indicate that the inhibitory effects of shikonin on mitochondrial biogenesis and metastasis in CAF-enhanced TNBC cells depend on inducing the phosphorylation of PGC-1α at Thr295, thereby promoting its ubiquitin-mediated degradation.

### PGC-1α is a promising biomarker of poor prognosis and a potential target of TNBC

**Fig. 7:**
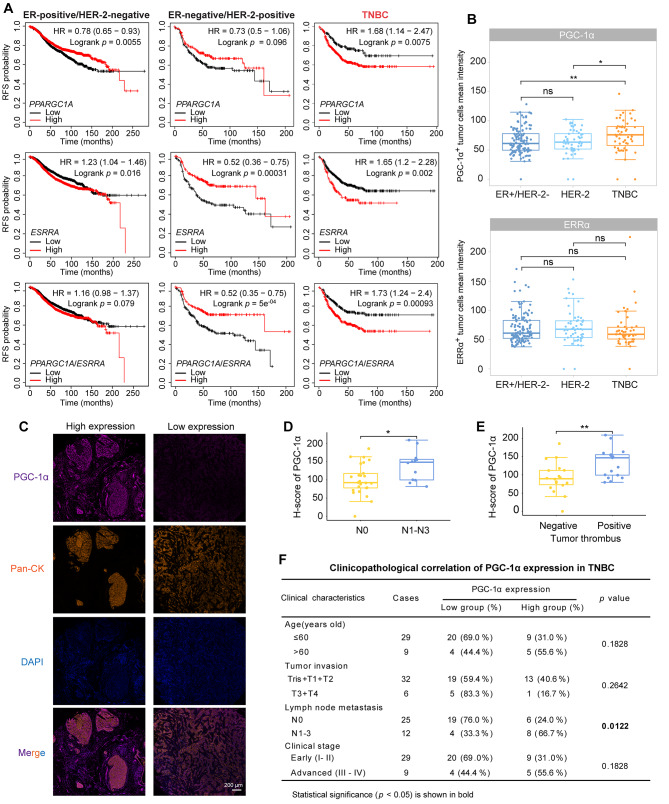
Correlation of high PGC-1α expression with poor prognosis in TNBC. (**A**) Associations between *PPARGC1A*, *ESSRA* and *PPARGC1A/ESSRA* mRNA levels and RFS in patients with different subtypes of breast cancer using Kaplan–Meier plotter analysis. *PPARGC1A* and *ESSRA* mRNA levels were categorized as high or low using the auto-select best cutoff mode. The *PPARGC1A*/*ESSRA* mRNA level was defined as high or low using the mean expression of selected genes. *PPARGC1A*: 219195_at; *ESSRA*: 203193_at. The log-rank test was employed. (**B**) The mean intensity of PGC-1α-positive and ERRα-positive tumor cells in different breast cancer subtypes. A Wilcoxon test was used. (**C**) Representative images of PGC-1α expression in TNBC tissues by multiplex immunofluorescence staining. Tissues were labeled with anti-PGC-1α antibody (purple), anti-Pan-CK antibody (orange), and DAPI (blue). (**D**) High PGC-1α expression correlation with lymph node metastasis status (*n* = 37). An unpaired two-sided *t*-test was used. (**E**) High PGC-1α expression correlation with tumor thrombus formation (*n* = 35). An unpaired two-sided *t*-test was used. (**F**) Clinicopathological correlation of PGC-1α expression in TNBC. χ^2^ test was used. ^*^p < 0.05, ^**^p < 0.01, ns: not significant

To determine the clinical significance of PGC-1α in TNBC treatment, we firstly examined the relationships between PGC-1α/ERRα levels and clinical outcomes in TNBC patients by Kaplan-Meier analysis. High levels of *PPARGC1A* correlated with shorter RFS in TNBC patients and longer RFS in ER-positive breast cancer patients, with no significant correlation observed in HER-2-positive breast cancer patients (Fig. 7A). Elevated *ESRRA* levels were associated with shorter RFS in ER-positive and TNBC patients, but longer RFS in HER-2-positive patients (Fig. 7A). In addition, elevated *PPARGC1*/*ESSRA* levels were associated with poor RFS in TNBC patients and improved RFS in HER-2-positive patients, with no significant association in ER-positive breast cancer patients (Fig. 7A). We next utilized an anti-Pan-CK antibody to distinguish between stromal cells (Pan-CK^−^) and cancer cells (Pan-CK^+^), to evaluate the expression of PGC-1α and ERRα in tumor cells from different breast cancer subtypes. It was found that the expression of PGC-1α was higher in TNBC cells than in ER-positive or HER-2-positive subtypes (Fig. 7B). However, there was no significant difference in the expression of ERRα in the three subtypes (Fig. 7B). These data indicate that, regarding the two proteins of the PGC-1α/ERRα axis, PGC-1α is particularly correlated with the poor prognosis of TNBC, consistent with the finding that PGC-1α, but not ERRα, is upregulated in TNBC cells in response to CAF-stimulation (Fig. 3A, B).

Subsequently, we further delved deeper into the prognostic role of PGC-1α in TNBC (Fig. 7C). PGC-1α expression levels in TNBC cells were higher in patients with N1–N3 lymph node metastasis compared with those without lymph node metastasis (N0) (Fig. 7D, and 7F). Additionally, patients with tumor thrombus exhibited higher PGC-1α expression in TNBC cells compared to those without tumor thrombus (Fig. 7E). However, PGC-1α expression in TNBC cells was not associated with tumor invasion, clinical stages or other clinical characteristics in TNBC (Fig. 7F). Collectively, these results indicate that high PGC-1α expression predicts a poor prognosis and high risk of metastasis in TNBC patients, which is a potential target of TNBC.

## Discussion

Cancer cells exhibit metabolic flexibility at various stages of the metastatic process [40]. In primary tumors, they predominantly rely on glycolytic metabolism for the swift production of ATP and the generation of cellular building blocks [41]. However, during invasive and metastasis, they prioritize survival over proliferation, shifting towards OXPHOS to produce ATP [42]. This mitochondrial metabolism is particularly advantageous for metastatic cancer cells, as it aids in overcoming challenges such as anoikis and metabolic defects due to the loss of matrix attachment [43], and facilitating their movement from hypoxic regions to distal tissues [5]. Active mitochondria also play a crucial role in enhancing cell motility [33]. Moreover, ATP from active mitochondria activates purinergic P2Y2 receptors in endothelial cells, leading to the opening of the endothelial barrier and enabling transendothelial migration of cancer cells, thus promoting extravasation [44]. Hence, targeting mitochondrial biogenesis presents a promising therapeutic approach to inhibit cell metastasis.

TNBC is the most challenging subtype due to its high rates of mitosis, migration, and invasion, leading to frequent metastasis [45]. Metastatic TNBC cells display increased mitochondrial biogenesis and function, characterized by elongated mitochondrial morphology and heightened expression of genes involved in oxidized metabolism [46]. CAFs are critical in promoting TNBC metastasis, inducing a shift to a mitochondrial metabolic phenotype in breast cancer cells, marked by increased TCA cycle activity [12]. Our study demonstrated that CAFs not only enhanced the migratory and invasive capabilities of TNBC cells but also increased mitochondrial biogenesis, resulting in a higher number of mitochondria with elongated shapes. Furthermore, CAFs contributed to the mitochondrial distribution of regional lamellipodia and the generation of ATP. However, shikonin effectively suppressed the mitochondrial biogenesis enhanced by CAFs, potentially blocking TNBC metastasis.

Mitochondria are recognized as a crucial target for drug discovery, with numerous drugs currently undergoing clinical trials, particularly for patients with metastatic cancer. Notable examples include electron transport chain inhibitor metformin, the TCA cycle inhibitor devimistat (CPI-613), and the glutaminolysis inhibitor telaglenastat (CB-839) [31, 47, 48]. However, these drugs often face challenges such as modest efficacy, insufficient accumulation in target tissues, and unexpected toxicity [47, 49]. Directly targeting mitochondria is controversial; hence, focusing on regulators of mitochondrial biogenesis seems promising. The PGC-1α/ERRα axis is a central regulator of mitochondrial biogenesis, playing a key role in enhancing the respiratory chain components and TCA enzymes [50]. Our study reveals that *PPARGCA1* mRNA levels positively correlated with ERRα-targeted genes involved in mitochondrial biogenesis, including *IDH3A*, *ATP5F1B*, *COX4I1*, and *CYCS*, in TNBC tissues rather than ER-positive or HER-2-positve subtypes; however, although these associations were significant (*p* < 0.05), the *R*-values of these correlations were low. Further investigations are therefore needed to explore the correlations between *PPARGCA1* and ERRα-targeted genes. In addition, we found that PGC-1α/ERRα axis correlated with unfavorable clinical outcome in in patients with TNBC, with CAFs stimulating its transcriptional activity, making it a potential target for inhibiting mitochondrial biogenesis in TNBC cells. Previous studies showed that ERRα alone does not affect mitochondrial genes, and it functions through its coregulators [51]. Consistently, we observed higher PGC-1α levels in TNBC cells compared with other breast cancer subtypes. CAFs stimulated PGC-1α/ERRα axis in TNBC cells by increasing PGC-1α expression, increased nuclear localization, and its function as a transcriptional coactivator. When PGC-1α was knockdown, the invasive ability promoted by CAF in TNBC cells was reduced. Moreover, PGC-1α is upregulated in breast cancer cells that metastasize to the lung or bone, driving their enhanced metastatic phenotype [52]. In this study, high PGC-1α expression was found to predict lymph node metastasis and tumor thrombus formation in TNBC patients, suggesting its pro-metastatic effects. It was shown that high levels of *PPARGC1*/*ERRSA* indicated a better prognosis in patients with HER-2-positive tumors. However, these results were obtained by analyzing whole tumor tissues, and further confirmation is warranted, specifically focusing on breast cancer cells within tumor tissues. Therefore, targeting PGC-1α represents a novel strategy to inhibit TNBC metastasis by restricting mitochondrial biogenesis. We discovered that shikonin downregulated PGC-1α expression, suppressing the PGC-1α/ERRα axis-regulated transcription function and their targeted genes involved in mitochondrial biogenesis.

PGC-1α is a short-lived protein, and its post-translational modification play a vital role in regulating its expression and activity [53]. Previous research has shown that its phosphorylation at Thr177 and Ser538 by AMP-activated protein kinase, or Thr262, Ser265, and Thr298 by p38 mitogen-activated protein kinase, enhances the activity and stability of PGC-1α [54, 55], while phosphorylation at Thr295 by GSK-3β promotes its degradation [38]. Consistently, our findings indicated that shikonin enhanced the phosphorylation of PGC-1α at Thr295 in CAF-stimulated TNBC cells by increasing the expression of GSK-3β and its interaction with PGC-1α. It has been demonstrated that GSK-3β mediated phosphorylation of PGC-1α leads to E3 ubiquitin ligase SCF^Cdc4^-dependent ubiquitylation and proteasomal degradation in primary embryonic mouse neurons [38]. However, in our study, NEDD4-1, but not SCF^Cdc4^, was identified as a binding partner of PGC-1α in TNBC cells treated with shikonin. NEDD4 E3 ubiquitin ligase family members have specific structural features that facilitate substrate recognition [56]. PGC-1α contains PPR motifs along with threonine phosphorylation sites, potentially allowing interaction with NEDD4-1 via WW domains. Our results confirmed that shikonin facilitated the interaction of NEDD4-1 and PGC-1α. When Thr295 was mutated to Ala295, the shikonin-induced interaction of GSK-3β and NEDD4-1 with PGC-1α and the ubiquitylation of PGC-1α were almost reversed. The inhibitory effects of shikonin on TNBC mitochondrial biogenesis and metastasis were observed in cells expressing PGC-1α^WT^ but not PGC-1α^A^^295^. This suggests that shikonin promotes the degradation of PGC-1α by increasing its phosphorylation at Thr295, leading to recognition by E3 ubiquitin ligase NEDD4-1 and subsequent ubiquitin-mediated proteolysis. These findings provide an additional phosphorylation-dependent regulatory mechanism by which GSK-3β/NEDD4-1 regulates PGC-1α degradation, suggesting that Thr295 is a central phosphorylation site for its degradation, with varying forms of E3 ubiquitin ligase recognition in different cellular context. Further investigation is needed to explore the specific domain of PGC-1α that interacts with GSK3β and NEDD4-1, as well as to fully understand the regulatory role of PGC-1α in mitochondrial metabolism and its impact on TNBC metastasis. Furthermore, we noticed that the expression of PGC-1α^A295^ was not increased by CAF-CM stimulation. However, CAF-CM still promoted mitochondrial biogenesis and a metastatic phenotype in PGC-1α^A295^-expressing TNBC cells. This suggests that CAF-CM may induce other post-translational modification, such as methylation or acetylation, to modulate PGC-1α activity, which warrants further investigation. Although no commercial drugs specifically targeting PGC-1α in cancer are currently available in clinics, several candidate compounds have shown promise in preclinical studies by inhibiting PGC-1α in cancer therapy [57]. More clinical trials are needed to explore these potential treatments in the future.

## Conclusions

**Fig. 8:**
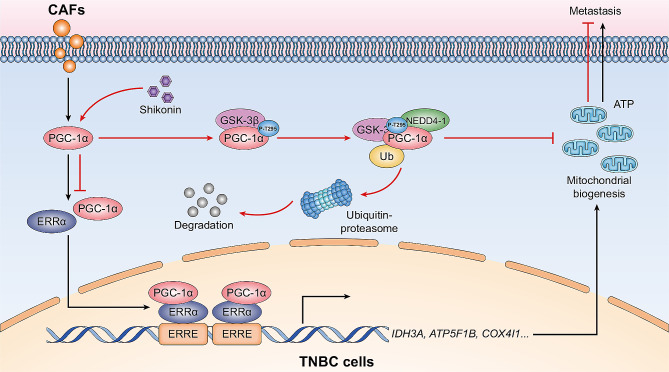
Schematic diagram of the inhibitory effects of shikonin on mitochondria biogenesis and metastasis in CAF-stimulated TNBC cells. Shikonin modulates the post-translation of PGC-1α by GSK-3β, increasing its phosphorylation at Thr295, which enhances NEDD4-1 recognition, leading to subsequent degradation by ubiquitin proteolysis. The inhibition of PGC-1α by shikonin results in restricted CAF-stimulated PGC-1α/ERRα axis-regulated mitochondrial biogenesis, ultimately reducing TNBC metastasis

Shikonin enhances the GSK-3β/NEDD4-1-mediated phosphorylation-depended ubiquitin-proteasome degradation of PGC-1α, thereby inhibiting PGC-1α/ERRα axis, which shows promise as a therapeutic strategy for inhibiting mitochondrial biogenesis and reducing TNBC metastasis (Fig. 8). Our study offers novel insights into therapeutic avenues for preventing TNBC metastasis through the inhibition of mitochondrial biogenesis. Additionally, we identified a novel mechanism of targeting PGC-1α for degradation through phosphorylated modification, which may provide opportunities for the discovery of new agents for TNBC therapy.

### Electronic supplementary material

Below is the link to the electronic supplementary material.

Supplementary Material 1

## Data Availability

All data are available from the corresponding author upon reasonable request.

## Abbreviations

α-SMA: Alpha-smooth muscle actin
ATPsynβ: ATP synthase β
BCA: Bicinchoninic acid
CAF: Cancer-associated fibroblast
ChIP: Chromatin immunoprecipitation
CHX: Cycloheximide
CM: Conditioned medium
COX: IV Cytochrome c oxidase subunit 4I1
Cyt* c*: Cytochrome c
DAPI: 6′ diamidino 2 phenylindole
ERRα: Estrogen-related receptor alpha
H&E: Hematoxylin and Eosin
IDH3A: Isocitrate dehydrogenase 3 (NAD+) α
IHC: Immunohistochemistry
LC-MS/MS: Liquid chromatography-mass spectrometry/mass spectrometry
NEDD4-1/NEDD4: Neural precursor cell expressed developmentally downregulated 4e1
OXPHOS: Oxidative phosphorylation
PAGE: Polyacrylamide gel electrophoresis
Pan-CK: Pan-cytokeratin
PGC-1α: Peroxisome-proliferator activated receptor coactivator 1α
PIC: Protease inhibitor cocktail
PMSF: Phenylmethanesulfonyl fluoride
PPR: Poly-proline rich
Q-PCR: Quantitative polymerase chain reaction
RFS: Relapse-free survival
RIPA: Radio immunoprecipitation assay
ROS: Reactive oxygen species
SDS: Sodium dodecyl sulfate
TCA: Tricarboxylic acid cycle
TME: Tumor microenvironment
TNBC: Triple-negative breast cancer
