# Supplementary material for: Shikonin blocks CAF-induced TNBC metastasis by suppressing mitochondrial biogenesis through GSK-3β/NEDD4-1 mediated phosphorylation-dependent degradation of PGC-1α
Source: J Exp Clin Cancer Res. 2024 Jun 27;43:180. doi: 10.1186/s13046-024-03101-z (PMC11210116; doi:10.1186/s13046-024-03101-z)
Supplement: Supplementary file 1 — Supplementary Material 1 [file 13046_2024_3101_MOESM1_ESM.docx]

**Supporting Information**

Shikonin blocks CAF-induced TNBC metastasis by suppressing mitochondrial biogenesis through GSK-3β/NEDD4-1 mediated phosphorylation-dependent degradation of PGC-1α

**Supplementary file includes:**

**Figure S1** Representative images of CAFs with immunohistochemical staining of α-SMA and fibronectin expression.

**Figure S2** Shikonin inhibits CAF-derived lactate-stimulated mitochondrial biogenesis in TNBC cells.

**Figure S3** Effect of shikonin on mitochondrial biogenesis in TNBC cells without CAF-stimulation.

**Figure S4** The role of PGC-1α in CAF-promoted invasive ability in TNBC cells.

**Figure S5** *PPARGC1A* mRNA levels in TNBC cells.

**Figure S6** Analysis of polyubiquitylated sites of PGC-1α in TNBC cells using LC-MS/MS.

**Figure S7** Shikonin enhances the interaction of PGC-1α with NEDD4-1 and GSK-3β in TNBC cells.

**Figure S8** Impact of shikonin on the body weight and organ histology.

**Table S1** Primer sequences for RT-PCR and ChIP.

**Table S2** Clinicopathological parameters of breast cancer specimens.

**Table S3** Clinicopathological parameters of TNBC specimens.

**Table S4** PGC-1α binding partners in TNBC cells after CAF-CM and shikonin treatment.

**Table S5** Hematological parameters for female NOD/SCID mice.

**Table S6** Functional parameters of heart, liver and kidney in NOD/SCID mice.

**Key reagents**

| MATERIAL | SOURCE | IDENTIFIER |
| --- | --- | --- |
| Regents |  |  |
| Shikonin | Solarbio | Cat#SS8500 |
| Z-Leu-Leu-Leu-al (MG132) | Aladdin | Cat#M126521 |
| Cycloheximide (CHX) | Med Chem Express | Cat#HY-12320 |
| Dulbecco's Modified Eagle Medium/Nutrient Mixture F-12 (DMEM/F-12) | Thermo Fisher Scientific | Cat#11330032 |
| Primary fibroblast basic medium | BeNa Culture Collection | Cat#BNCC357956 |
| Fibroblast culture additive | BeNa Culture Collection | Cat#BNCC357703 |
| Fetal Bovine Serum | Thermo Fisher Scientific | Cat#10099141 |
| Penicillin-Streptomycin Solution | Wisent Corporation | Cat#450-201-CL |
| Dispase | Gibco | Cat#17105041 |
| Type I collagenase | Sigma | Cat#SCR103 |
| Trypsin (with 0.25% EDTA) | Thermo Fisher Scientific | Cat#25200056 |
| CellTracker™ Green (5-chloromethylfluorescein diacetate) | Aladdin | Cat#[C131098](https://www.aladdin-e.com/zh_cn/c131098.html) |
| Matrigel^®^ Matrix Basement Membrane | Corning | Cat#356234 |
| Matrigel^®^ Basement Membrane Matrix Growth Factor Reduced Phenol-Red Free | Corning | Cat#356231 |
| Hematoxylin and Eosin (H&E) staining regents | Beyotime | Cat#C0105S |
| ActinGreen^™^ 488 ReadyProbes^™^ reagent | Thermo Fisher Scientific | Cat#R37110 |
| Poly (2-hydroxyethyl methacrylate), poly-HEMA | Sigma | Cat#P3932 |
| MitoTracker^™^ Green FM | Thermo Fisher Scientific | Cat#M7514 |
| MitoTracker^™^ Deep Red FM | Thermo Fisher Scientific | Cat#M22426 |
| 2-(4-Amidinophenyl)-6-indolecarbamidine dihydrochloride (DAPI) | Solarbio | Cat#C0060 |
| Hoechst 33342 | Beyotime | Cat#C1028 |
| 2',7'-Dichlorodihydrofluorescein diacetate  (H2DCFDA） | Med Chem Express | Cat#HY-D0940 |
| MitoSOX^™^ Red Mitochondrial Superoxide Indicator | Thermo Fisher Scientific | Cat#M36008 |
| Radio Immunoprecipitation Assay (RIPA) lysis buffer | Beyotime | Cat#P0013K |
| Digitonin | Sigma | Cat#D5628 |
| Phenylmethanesulfonyl fluoride (PMSF) | Beyotime | Cat#ST505 |
| ProteinSafe Protease Inhibitor Cocktail, EDTA-free (100×, PIC) | TransGen Biotech | Cat#DI101 |
| NativePAGE^™^ 4× Sample Buffer | Thermo Fisher Scientific | Cat#BN20032 |
| NativePAGE^™^ 5% G-250 Sample Additive | Thermo Fisher Scientific | Cat#BN2004 |
| NativePAGE^™^ 4-16% Bis-Tris Gel | Thermo Fisher Scientific | Cat#BN1002BOX |
| NativePAGE^™^ 20× Running Buffer | Thermo Fisher Scientific | Cat#BN2001 |
| NativePAGE^™^ 20× Cathode Buffer Additive | Thermo Fisher Scientific | Cat#BN2002 |
| NuPAGE Transfer Buffer (20×) | Thermo Fisher Scientific | Cat#NP0006 |
| Bovine Serum Albumin (BSA) | Sigma | Cat#A-6003 |
| Immobilon Western Chemiluminescent HRP Substrate | Millipore | Cat#WBKLS0500 |
| Olive oil solvent | Sigma | Cat#1478265 |
| pGL3-3×ERRE luciferase reporter plasmid | Addgene | Cat#37851 |
| pRL-TK vector plasmid | Promega | Cat#E2241 |
| Antibodies | SOURCE | IDENTIFIER |
| PGC-1α Mouse Monoclonal Antibody | Proteintech | Cat#66369-1-Ig |
| PGC-1α Mouse Monoclonal Antibody  (Multiplex immunofluorescence) | Sigma | Cat#ST1202 |
| Pan-cytokeratin (Pan-CK) Mouse Monoclonal Antibody | Cell Signaling Technology | Cat#4545 |
| Estrogen Related Receptor alpha (ERRα) Rabbit Monoclonal Antibody | Abcam | Cat#ab76228 |
| EpCAM Rabbit Polyclonal Antibody | Affinity | Cat#DF6311 |
| Oxidative Phosphorylation Mouse Cocktail Antibody | Thermo Fisher Scientific | Cat#45-8099 |
| ATPase Rabbit Polyclonal Antibody | Proteintech | Cat#17247-1-AP |
| COXIV Rabbit Polyclonal Antibody | Proteintech | Cat#11242-1-AP |
| Cytochrome c (Cyt c) Mouse Monoclonal Antibody | Proteintech | Cat#66264-1-Ig |
| IDH3A Rabbit Polyclonal Antibody | Proteintech | Cat#15909-1-AP |
| mtTFA Rabbit Polyclonal Antibody | Proteintech | Cat#22586-1-AP |
| TIM22 Rabbit Polyclonal Antibody | Proteintech | Cat#14927-1-AP |
| TOM20 Rabbit Polyclonal Antibody | Proteintech | Cat#11802-1-AP |
| Ubiquitin (P4D1) Mouse Monoclonal Antibody | Cell Signaling Technology | Cat#3936 |
| β-Actin (4D3) Mouse Monoclonal Antibody | Bio-World | Cat#BS6007M |
| Lamin B1 (L75) Rabbit Polyclonal Antibody | Bio-World | Cat#BS3547 |
| LDHA Rabbit Polyclonal Antibody | Proteintech | Cat#21799-1-AP |
| Smooth muscle actin Rabbit Polyclonal Antibody | Proteintech | Cat#14395-1-AP |
| Fibronectin Rabbit Polyclonal Antibody | Proteintech | Cat#15613-1-AP |
| Goat Polyclonal Secondary Antibody to Rabbit IgG-H&L (Alexa Fluor^®^ 647) | Abcam | Cat#ab150079 |
| Goat Polyclonal Secondary Antibody to Rabbit IgG-H&L (Alexa Fluor^®^ 488) | Abcam | Cat#ab150077 |
| Goat Polyclonal Secondary Antibody to Mouse IgG-H&L (Alexa Fluor^®^ 488) | Abcam | Cat#ab150113 |
| Goat anti-Mouse IgG (H+L)-HRP | Bio-World | Cat#BS12478 |
| Goat anti-Rabbit IgG (H+L)-HRP | Bio-World | Cat#BS13278 |
| Kits | SOURCE | IDENTIFIER |
| SABC-HRP Kit with Anti-Rabbit IgG | Beyotime | Cat#P0615 |
| Annexin V-FITC/PI Apoptosis Detection Reagent | KeyGen BioTECH | Cat#KGA107 |
| CytoSelect^™^ 96-Well Anoikis Assay Kit | Cell Biolabs | Cat#CBA-081 |
| ATP Determination Kit | Thermo Fisher Scientific | Cat#A22066 |
| Nuclear and Cytoplasmic Protein Extraction Kit | Beyotime | Cat#P0028 |
| Cell mitochondria Isolation Kit | Beyotime | Cat#C3601 |
| Pierce™ BCA Protein Assay Kit | Thermo Fisher Scientific | Cat#23227 |
| Dual-Luciferase® Reporter Assay System | Promega | Cat#E1910 |
| Chromatin Immunoprecipitation (ChIP) Kit | BersinBio | Cat#Bes5001 |
| MiniBEST Universal Genomic DNA Extraction Kit | TaKaRa | Cat#9765 |
| Human Mitochondrial DNA Monitoring Primer Set | TaKaRa | Cat#7246 |
| TB Green^®^ Premix Ex Taq^™^II (Tli RNaseH Plus) | TaKaRa | Cat#RR820A |
| MiniBEST Universal RNA Extraction kit | TaKaRa | Cat#9767 |
| PrimeScript^™^ RT reagent Kit with gDNA Eraser (Perfect Real Time） | TaKaRa | Cat#RR047A |
| Immunoprecipitation Kit (Protein A/G Plus Agarose) | Sangon Biotech | Cat#C600689-0020 |
| Immunohistochemical stain detection kit | Zhongshan Golden Bridge Biotechnology | Cat#PV-9000 |
| L-Lactate Assay Kit | Abcam | Cat#ab65330 |
| PANO 7-plex IHC Kit | Panovue | Cat#10004100100 |
| Lactate Dehydrogenase (LDH) Assay Kit | Nanjing Jiancheng Bioengineering Institute | Cat#A020-2-1 |
| Creatine Kinase (CK) Assay Kit (Colorimetric Method) | Nanjing Jiancheng Bioengineering Institute | Cat#A032-1-1 |
| Albumin Assay Kit Instruction | Nanjing Jiancheng Bioengineering Institute | Cat#A028-2-1 |
| Alanine Transarninase/Glutamate Pyruvate Transaminase (ALT/GPT) Assay Kit (Reitman-Frankel’s Method) | Nanjing Jiancheng Bioengineering Institute | Cat#C009-2-1 |
| Aspartate Aminotransferase/Glutamic Oxaloacetic Transaminase (AST/GOT) Assay Kit (Reitman-Frankel’s Method) | Nanjing Jiancheng Bioengineering Institute | Cat#C010-2-1 |
| Blood Urea Nitrogen Assay Kit Instruction | Nanjing Jiancheng Bioengineering Institute | Cat#C013-2-1 |
| Creatinine Assay Kit Assay Kit Instruction | Nanjing Jiancheng Bioengineering Institute | Cat#C011-2-1 |
| Uric Acid Assay Kit Instruction | Nanjing Jiancheng Bioengineering Institute | Cat#C012-2-1 |

**Cell Culture conditions**

| Cell lines | Cell type | Culture conditions |
| --- | --- | --- |
| MDA-MB-231 | TNBC | DMEM/F-12 medium supplemented with 10% fetal bovine serum (FBS) and penicillin (100 U mL^-1^)-streptomycin (100 μg mL^-1^) solution. |
| MDA-MB-468 | TNBC |  |
| CAF | Cancer-associated fibroblasts | Primary fibroblast basic medium supplemented with 1% fibroblast supplement and 10% FBS without light. |
| WI-38 | Normal fibroblast cells | EMEM medium supplemented with 10% FBS and penicillin (100 U mL^-1^)-streptomycin (100 μg mL^-1^) solution. |
| MCF-10A | Normal breast epithelial cell | 1:1 mixture of Ham’s F-12 medium and Dulbecco’s modified Eagle’s medium with 5% horse serum, insulin (10 μg mL^-1^), EGF (20 ng mL^-1^), hydrocortisone (0.5 μg mL^-1^), cholera enterotoxin (0.1 μg mL^-1^) and penicillin (100 U mL^-1^)-streptomycin (100 μg mL^-1^) solution. |
| MCF-12A | Normal breast epithelial cell |  |
| MCF-7 | ER-positive breast cancer cells | DMEM/F-12 medium supplemented with 10% FBS and penicillin (100 U mL^-1^)-streptomycin (100 μg mL^-1^) solution. |
| T-47D | ER-positive breast cancer cells |  |
| SK-BR-3 | HER-2-postive breast cancer cells | RPMI-1640 medium supplemented with 10% FBS and penicillin (100 U mL^-1^)-streptomycin (100 μg mL^-1^) solution. |


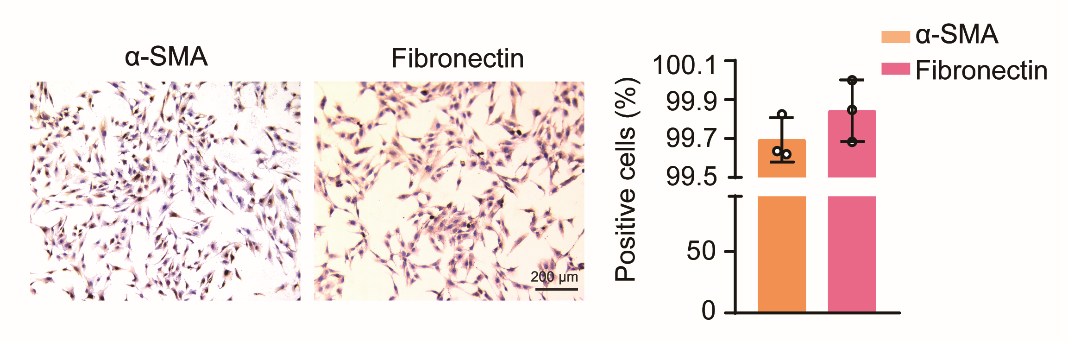


**Fig. S1** Representative images of CAFs with immunohistochemical staining of α-SMA and fibronectin expression. Immunohistochemical staining of CAFs was conducted using a SABC-HRP kit with anti-α-SMA (1:500) and anti-fibronectin (1:500) antibodies. The α-SMA-positive and fibronectin-positive cells quantified by ImageJ software. (magnification, ×100; scale bars, 200 μm; *n* = 3).


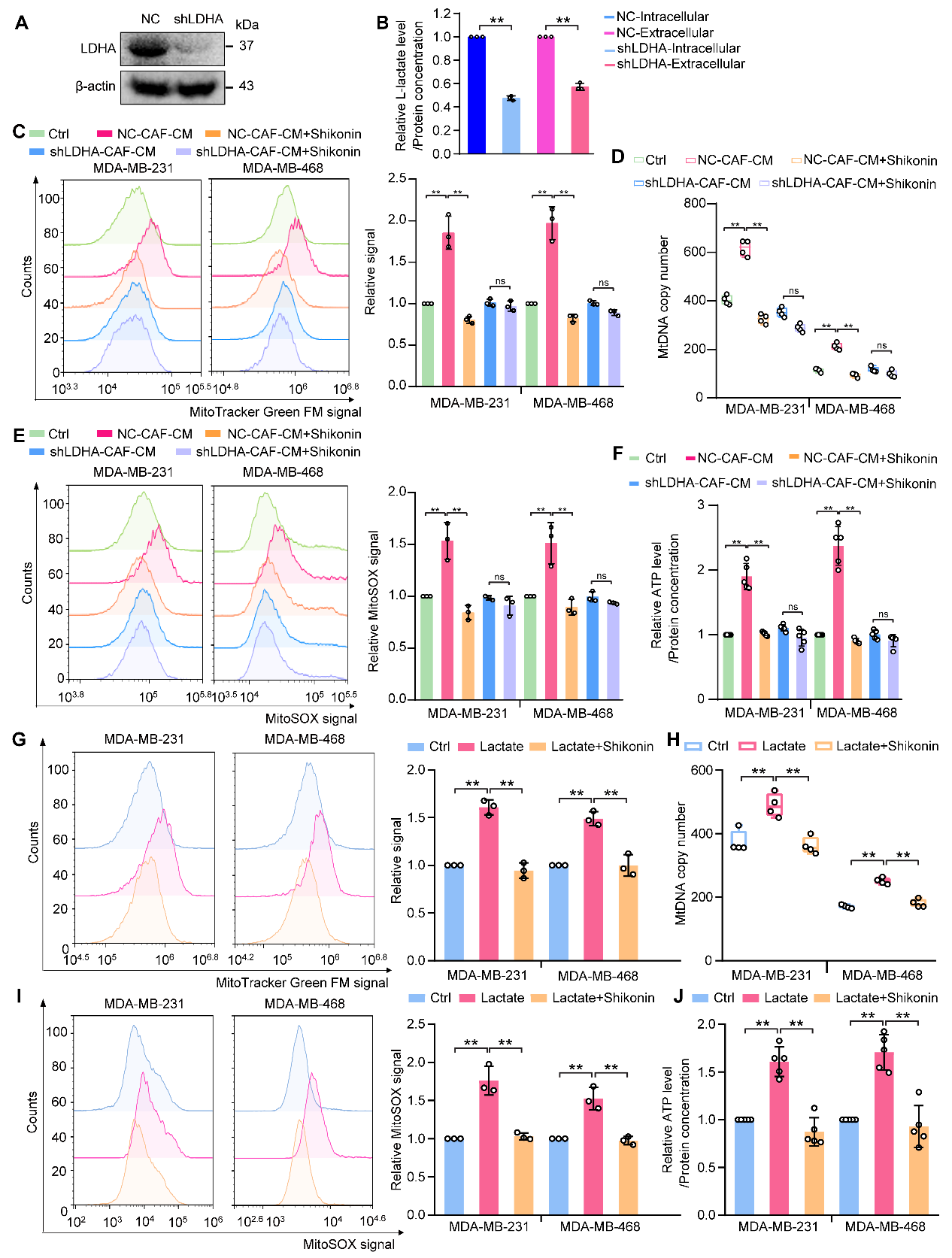


**Fig. S2** Shikonin inhibits CAF-derived lactate-stimulated mitochondrial biogenesis in TNBC cells. CAFs were transfected with shNC or shLDHA and then CAF-CM was used to stimulate TNBC cells: (B)-(F). **(A)** The LDHA expression in CAFs. **(B)** Intracellular and extracellular levels of L-lactate in CAFs were measured using an L-lactate assay kit. Lactate levels were normalized to the protein concentration (*n* = 3). **(C)** Detection of mitochondria in TNBC cells using MitoTracker Green FM staining and flow cytometry (*n* = 3). **(D)** Evaluation of mtDNA levels in TNBC cells (*n* = 4). **(E)** Assessment of mitochondrial ROS levels in TNBC cells using MitoSOX labeling flow cytometry (*n* = 3). **(F)** Calculation of ATP content in TNBC cells from cellular extracts using an ATP detection kit. Intracellular ATP levels were normalized to the protein concentration (*n* = 5). **(G)** Mitochondria were detected using MitoTracker Green staining and quantified by flow cytometry (*n* = 3). **(H)** mtDNA levels were evaluated relative to levels of ND1 and ND5, compared with SLCO2B1 and SERPINA1 as controls (*n* = 4). **(I)** Mitochondrial ROS levels were determined using MitoSOX labeling and measured by flow cytometry (*n* = 3). **(J)** ATP content in cellular extracts was calculated using an ATP detection kit, and intracellular ATP levels were normalized to the protein concentration (*n* = 5). Data are presented as mean ± SD.***p* < 0.01, ns: not significant.


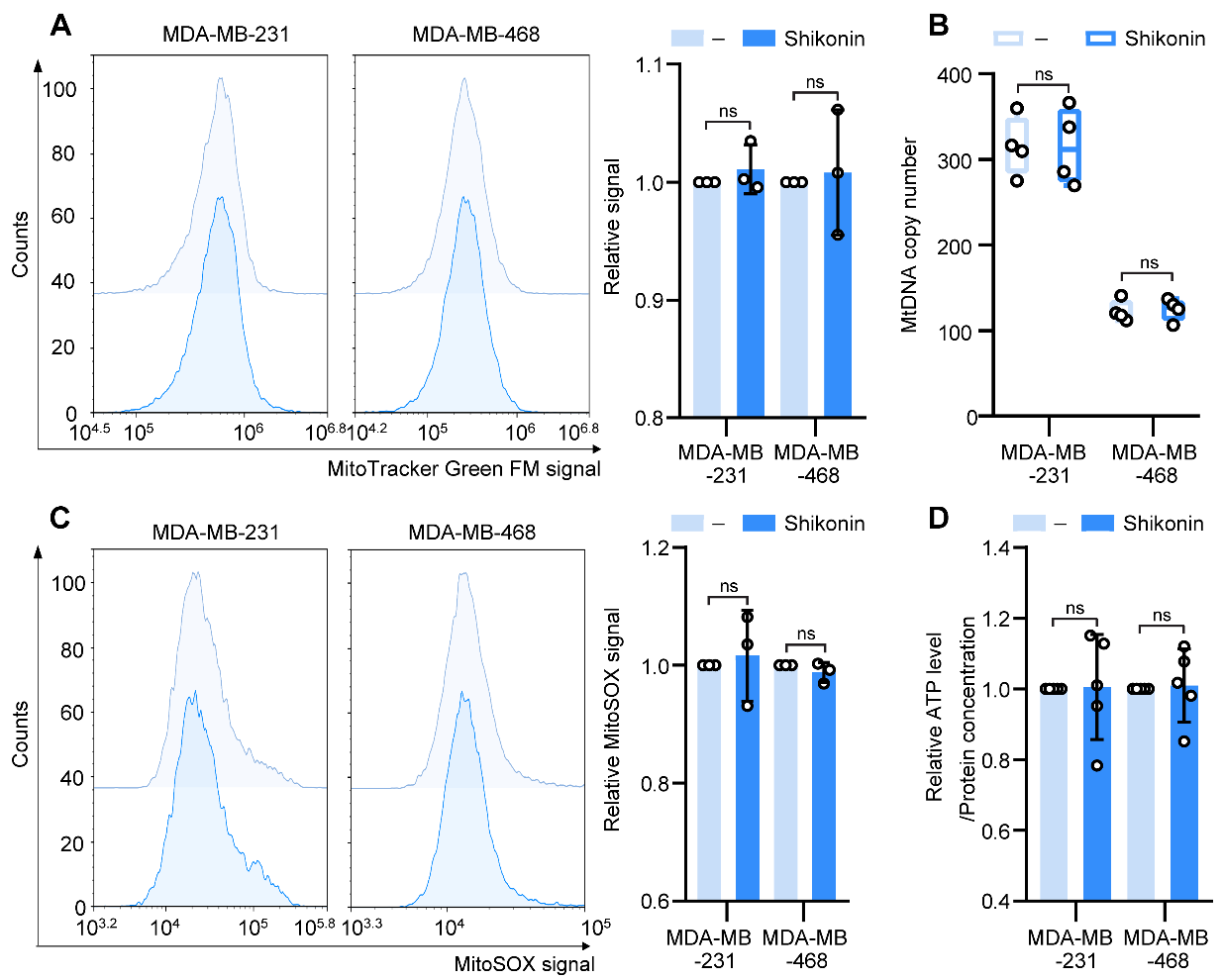


**Fig. S3** Effect of shikonin on mitochondrial biogenesis in TNBC cells without CAF-stimulation. **(A)** Mitochondria were visualized using MitoTracker Green staining and quantified by flow cytometry (*n* = 3). **(B)** mtDNA levels were assessed relative to ND1 and ND5, with SLCO2B1 and SERPINA1 serving as controls (*n* = 4). **(C)** Mitochondrial ROS levels were assessed using MitoSOX labeling and measured by flow cytometry (*n* = 3). **(D)** ATP content in cellular extracts was quantified using an ATP detection kit, and intracellular ATP levels were normalized to the protein concentration (*n* = 5). Data are presented as mean ± SD. ns: not significant.


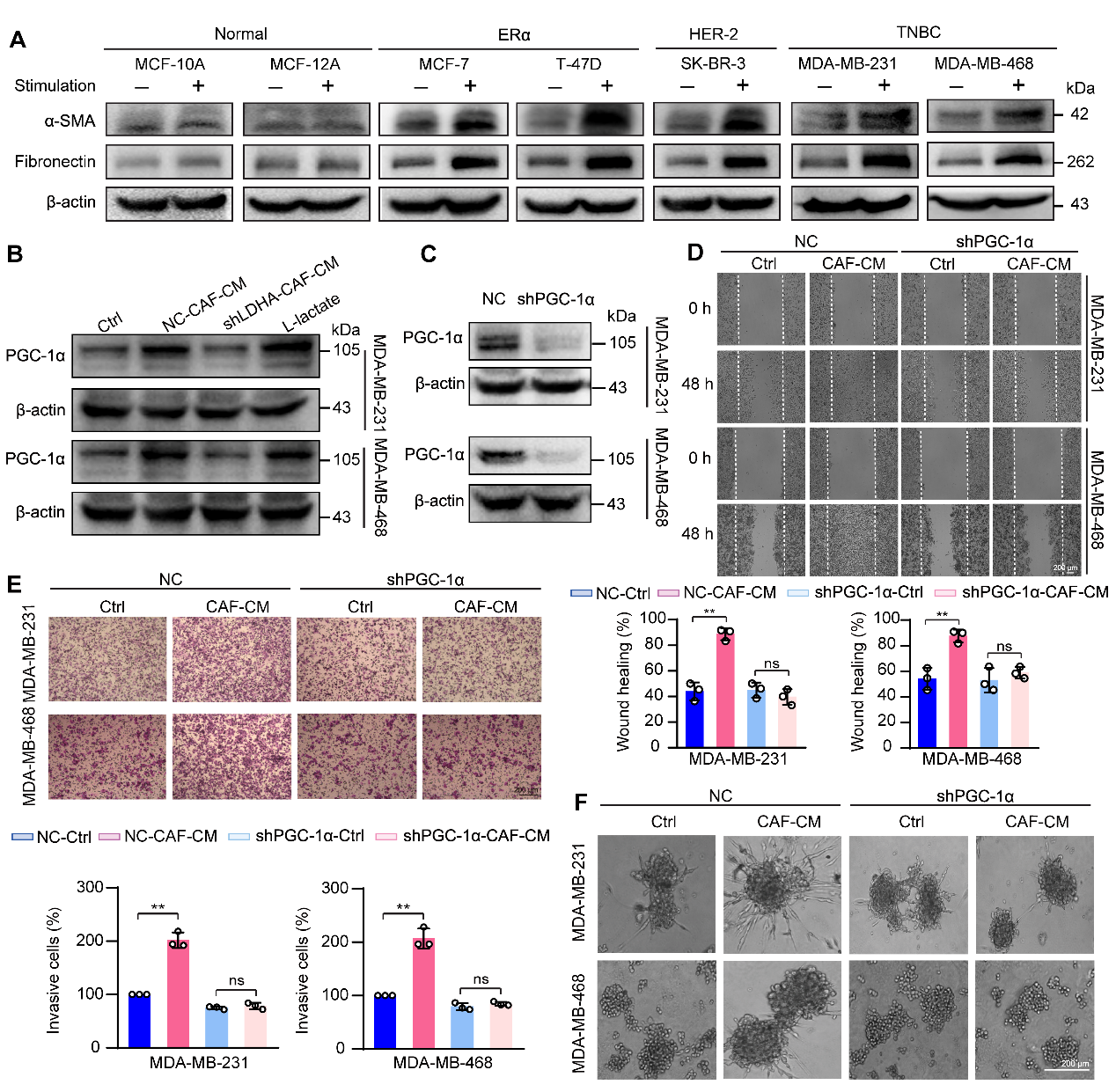


**Fig. S4** The role of PGC-1α in CAF-promoted invasive ability in TNBC cells. **(A)** The expression of CAF-related makers (α-SMA and fibronectin) in WI-38 cells stimulated by different breast cancer subtype cells. The culture medium from different subtypes breast cancer cells was used to activate WI-38 cells for 48 h to stimulate WI-38 cells to be transformed as CAFs. **(B)** CAFs were transfected with shNC and shLDHA, then CAF-CM were obtained to stimulate TNBC cells. Western blot analyses of PGC-1α expression. TNBC cells with or without PGC-1α knockdown were treated with CAF-CM for 48 h: (C)-(F). **(C)** The expression of PGC-1α in TNBC cells. **(D)** Cell migration was measured by wound-healing assay. Images were captured at 0 and 48 h post-wounding (magnification, ×50; scale bars, 200 μm, *n* = 3). **(E)** Cell invasion was measured by Transwell assay. TNBC were stimulated with CAF-CM for 48 h, and then inoculated in the upper compartment, and media containing 10% FBS was added into the lower compartment for 24 h-incubation. Images were captured (magnification, 100×; scale bars, 200 μm, *n* = 3). **(F)** TNBC cells with or without PGC-1α knockdown were grown in Matrigel. Cells were treated with CAF-CM for 3 days (magnification, ×100; scale bars, 200 μm). Data are presented as mean ± SD. ***p* < 0.01, ns: not significant.


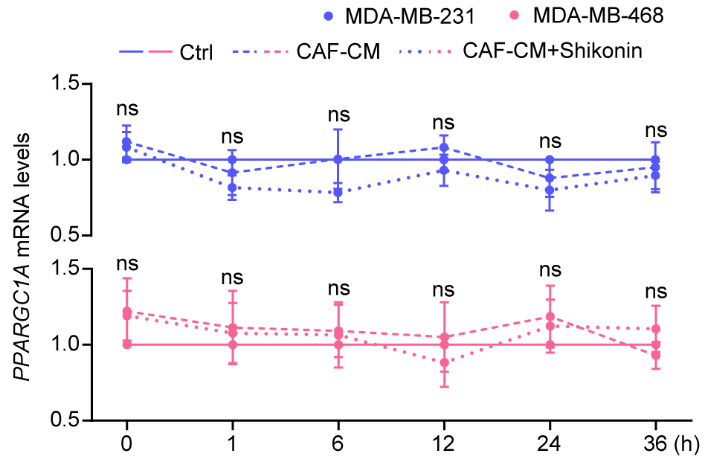


**Fig. S5** *PPARGC1A* mRNA levels in TNBC cells. The results are presented as fold change compared to the control (*n* = 3). *ACTB* was used as the reference gene. ns: not significant.


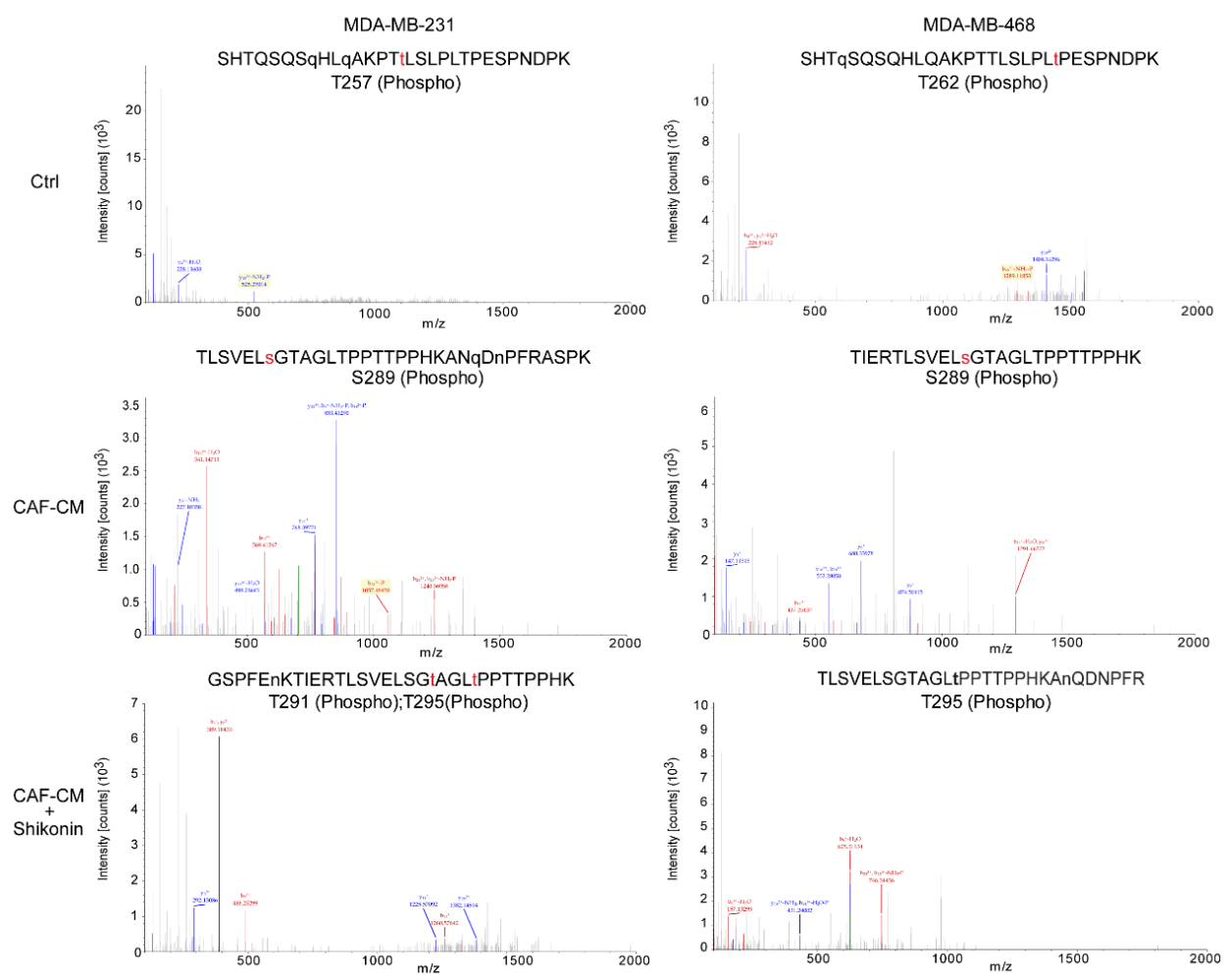


**Fig. S6** Analysis of polyubiquitylated sites of PGC-1α in TNBC cells using LC-MS/MS.


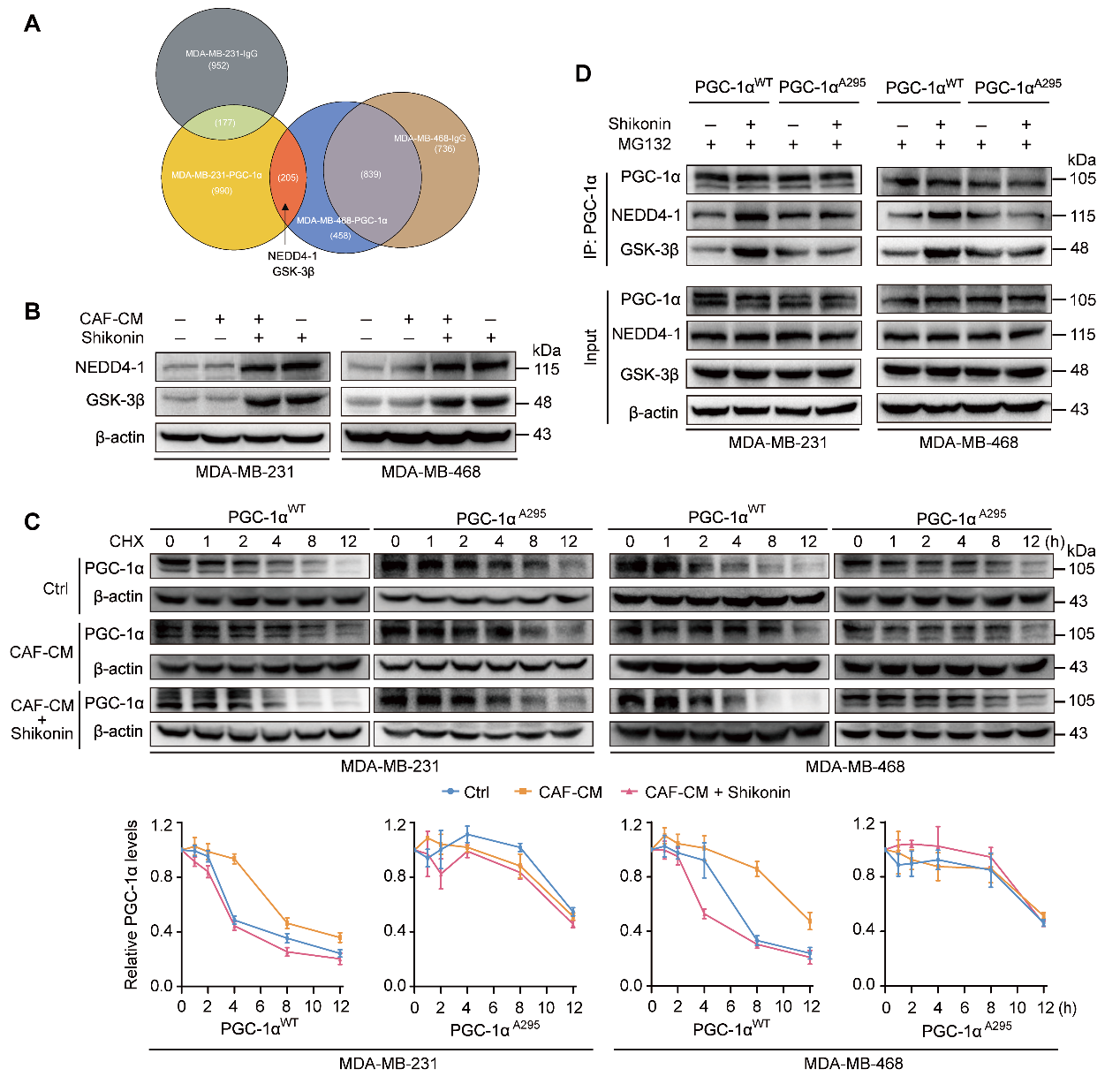
**Fig. S7** Shikonin enhances the interaction of PGC-1α with NEDD4-1 and GSK-3β in TNBC cells. **(A)** The Venn diagram illustrates the binding partners of PGC-1α analyzed by immunoprecipitation using an anti-PGC-1α antibody or anti-IgG antibody. The binding partners of PGC-1α or IgG were identified by LC-MS/MS. **(B)** Expression levels of NEDD4-1 and GSK-3β in TNBC cells. TNBC cells were treated with CAF-CM in the presence or absence of shikonin (2 μM) for 48 h. **(C)** The half-life of PGC-1α^WT^ or PGC-1α^A295^ protein in CAF-stimulated TNBC cells. Cells were treated with CAF-CM in the presence or absence of shikonin under cycloheximide (CHX, 6 μM) treatment as indicated time points and harvested for western blotting (*n* = 3). **(D)** Mutation of PGC-1α at Thr295 reversed the inhibitory effects of shikonin on the interaction of PGC-1α with NEDD4-1 and GSK-3β in TNBC cells. TNBC cells were transfected with shPGC-1α vectors followed with PGC-1α^WT^ and PGC-1α^Α295^ plasmids, and then treated with CAF-CM in the presence or absence of shikonin (2 μM) for 48 h.


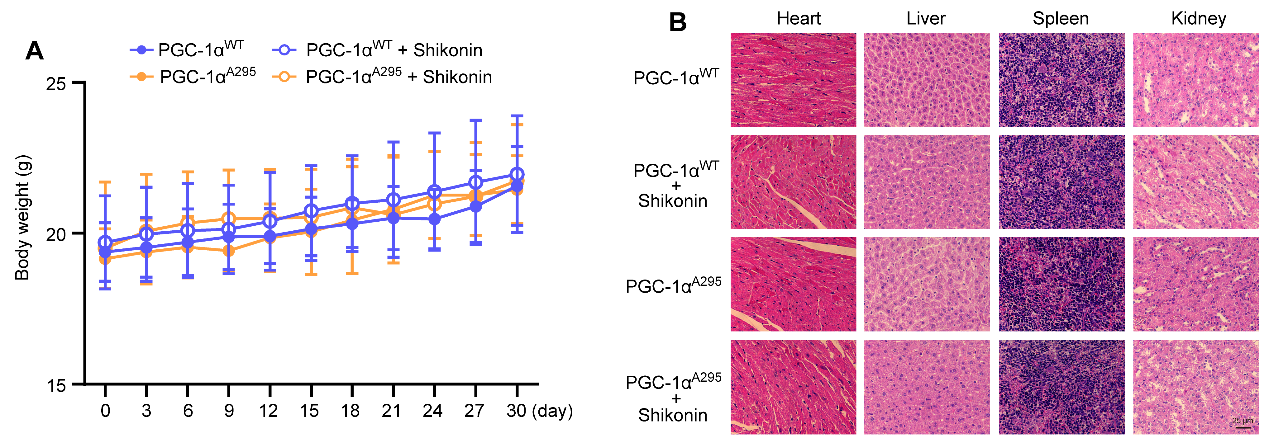


**Fig. S8** Impact of shikonin on the body weight and organ histology. **(A)** Body weight comparison between mice with or without shikonin treatment (*n* = 10). **(B)** Representative H&E staining of heart, liver, spleen, and kidney sections (magnification, ×400; scale bars, 25 μm, *n* = 10). Results are presented as mean ± SD.

**Table S1** Primer sequences for RT-PCR and ChIP.

| Name | 5’-3’ sequence | Reference | Type |
| --- | --- | --- | --- |
| *PPARGC1A* forward | CAC CAG CCA ACA CTC AGC TA | [1] | RT-PCR |
| *PPARGC1A* reverse | GTG TGA GGA GGG TCA TCG TT |  |  |
| *CYCS* forward | GGC TGC AGT GTA GCT GTG AT | [2] | RT-PCR |
| *CYCS* reverse | GAT GGA GTT TCC TTT ATC TGT TGC |  |  |
| *IDH3A* forward | TGC CCA GAC TGA AGA GAC CT | [3] | RT-PCR |
| *IDH3A* reverse | TTT CCC TTT TAG GTC CCA CGA |  |  |
| *COX4I1* forward | TTT CAC CGC GCT CGT TAT | [4] | RT-PCR |
| *COX4I1* reverse | CTT CAT GTC CAG CAT CCT CTT |  |  |
| *ATP5F1B* forward | TGG TGG TGC TGG ACT TGG | [5] | RT-PCR |
| *ATP5F1B* forward | GCC TGG GTG AAG CGA AAG |  |  |
| *ESRRA* forward | GTG ACC TTC ATT CGG TCA CCG CA | [6] | ChIP |
| *ESRRA* forward | CAC TGG AAG TAA GCC AGT GGC GT |  |  |
| *ACTB* forward | AGA AGG CTG GGG CTC ATT TG | [1] | RT-PCR |
| *ACTB* reverse | AGG GGC CAT CCA CAG TCT TC |  |  |

**References**

1. Zu Y, Chen XF, Li Q, Zhang ST, Si LN. PGC-1α activates SIRT3 to modulate cell proliferation and glycolytic metabolism in breast cancer. Neoplasma. 2021;68(2):352-61.

2. Panji M, Behmard V, Zare Z, Malekpour M, Nejadbiglari H, Yavari S, et al. Synergistic effects of green tea extract and paclitaxel in the induction of mitochondrial apoptosis in ovarian cancer cell lines. Gene. 2021;787:145638.

3. Liu X, Qiao Y, Ting X, Si W. Isocitrate dehydrogenase 3A, a rate-limiting enzyme of the TCA cycle, promotes hepatocellular carcinoma migration and invasion through regulation of MTA1, a core component of the NuRD complex. Am J Cancer Res. 2020;10(10):3212-29.

4. Douiev L, Miller C, Ruppo S, Benyamini H, Abu-Libdeh B, Saada A. Upregulation of COX4-2 via HIF-1α in Mitochondrial COX4-1 Deficiency. Cells. 2021;10(2).

5. Yang J, Xiao X, Li R, Li Z, Deng M, Zhang G. Hypermethylation of CpG sites at the promoter region is associated with deregulation of mitochondrial ATPsyn-β and chemoresistance in acute myeloid leukemia. Cancer Biomark. 2016;16(1):81-8.

6. Laganière J, Tremblay GB, Dufour CR, Giroux S, Rousseau F, Giguère V. A polymorphic autoregulatory hormone response element in the human estrogen-related receptor alpha (ERRalpha) promoter dictates peroxisome proliferator-activated receptor gamma coactivator-1alpha control of ERRalpha expression. J Biol Chem. 2004;279(18):18504-10.

**Table S2** Clinicopathological parameters of breast cancer specimens.

| Parameters | Number of cases (%) |
| --- | --- |
| **Gender** |  |
| Female | 230 (100.0) |
| **Age (years)** |  |
| ≤60 | 30 (13.04) |
| ＞60 | 200 (86.96) |
| **Pathological type** |  |
| Invasive ductal carcinoma | 208 (90.43) |
| Others | 22 (10.58) |
| **Breast cancer subtype** |  |
| ER-positive | 127 (55.22) |
| HER-2-positive | 52 (22.61) |
| TNBC | 51 (22.17) |

**Table S3** Clinicopathological parameters of TNBC specimens.

| Parameters | Number of cases (%) |
| --- | --- |
| **Gender** |  |
| Female | 38 (100.0) |
| **Age (years)** |  |
| ≤60 | 29 (76.32) |
| ＞60 | 9 (23.68) |
| **Pathological type** |  |
| Invasive ductal carcinoma | 34 (89.47) |
| Others | 4 (10.53) |
| **T classification** |  |
| Tis | 4 (10.53) |
| T_1_ | 18 (47.37) |
| T_2_ | 10 (26.32) |
| T_3_ | 3 (7.89) |
| T_4_ | 3 (7.89) |
| **N classification** |  |
| N_0_ | 25 (65.79) |
| N_1_ | 8 (21.05) |
| N_2_ | 3 (7.89) |
| N_3_ | 1 (2.63) |
| Information missing | 1 (2.63) |
| **N classification** |  |
| M_0_ | 37 (97.37) |
| M_1_ | 1 (2.63) |
| **Clinical stage** |  |
| Ⅰ～II | 29 (76.32) |
| III～Ⅳ | 9 (23.68) |
| **Thrombus formation** |  |
| Yes | 17 (44.74) |
| No | 18 (47.47) |
| Information missing | 3 (7.89) |

**Table S4** PGC-1α binding partners in TNBC cells after CAF-CM and shikonin treatment.

| **Number** | **Description** |
| --- | --- |
| 1 | Bcl-2-associated transcription factor 1 OS=Homo sapiens OX=9606 GN=BCLAF1 PE=1 SV=2 |
| 2 | Ankyrin repeat and KH domain-containing protein 1 OS=Homo sapiens OX=9606 GN=ANKHD1 PE=1 SV=1 |
| 3 | Probable ATP-dependent RNA helicase DDX5 OS=Homo sapiens OX=9606 GN=DDX5 PE=1 SV=1 |
| 4 | Protein spire homolog 1 OS=Homo sapiens OX=9606 GN=SPIRE1 PE=1 SV=3 |
| 5 | Glycogen synthase kinase-3 beta OS=Homo sapiens OX=9606 GN=GSK3B PE=1 SV=2 |
| 6 | Signal recognition particle 54 kDa protein OS=Homo sapiens OX=9606 GN=SRP54 PE=1 SV=1 |
| 7 | E3 ubiquitin-protein ligase TRIM33 OS=Homo sapiens OX=9606 GN=TRIM33 PE=1 SV=3 |
| 8 | Succinate--CoA ligase [GDP-forming] subunit beta, mitochondrial OS=Homo sapiens OX=9606 GN=SUCLG2 PE=1 SV=2 |
| 9 | Ataxin-2-like protein OS=Homo sapiens OX=9606 GN=ATXN2L PE=1 SV=2 |
| 10 | Importin-7 OS=Homo sapiens OX=9606 GN=IPO7 PE=1 SV=1 |
| 11 | Signal recognition particle subunit SRP68 OS=Homo sapiens OX=9606 GN=SRP68 PE=1 SV=2 |
| 12 | Tubulin alpha-1C chain OS=Homo sapiens OX=9606 GN=TUBA1C PE=1 SV=1 |
| 13 | Protein cornichon homolog 4 OS=Homo sapiens OX=9606 GN=CNIH4 PE=1 SV=1 |
| 14 | RalBP1-associated Eps domain-containing protein 1 OS=Homo sapiens OX=9606 GN=REPS1 PE=1 SV=3 |
| 15 | Tyrosine-protein phosphatase non-receptor type 2 OS=Homo sapiens OX=9606 GN=PTPN2 PE=1 SV=2 |
| 16 | Vasodilator-stimulated phosphoprotein OS=Homo sapiens OX=9606 GN=VASP PE=1 SV=3 |
| 17 | Flotillin-2 OS=Homo sapiens OX=9606 GN=FLOT2 PE=1 SV=2 |
| 18 | Pachytene checkpoint protein 2 homolog OS=Homo sapiens OX=9606 GN=TRIP13 PE=1 SV=2 |
| 19 | DnaJ homolog subfamily A member 1 OS=Homo sapiens OX=9606 GN=DNAJA1 PE=1 SV=2 |
| 20 | Interleukin enhancer-binding factor 2 OS=Homo sapiens OX=9606 GN=ILF2 PE=1 SV=2 |
| 21 | Tight junction protein ZO-2 OS=Homo sapiens OX=9606 GN=TJP2 PE=1 SV=2 |
| 22 | Activating signal cointegrator 1 complex subunit 1 OS=Homo sapiens OX=9606 GN=ASCC1 PE=1 SV=1 |
| 23 | Obg-like ATPase 1 OS=Homo sapiens OX=9606 GN=OLA1 PE=1 SV=2 |
| 24 | Tyrosine-protein phosphatase non-receptor type 1 OS=Homo sapiens OX=9606 GN=PTPN1 PE=1 SV=1 |
| 25 | Proliferation-associated protein 2G4 OS=Homo sapiens OX=9606 GN=PA2G4 PE=1 SV=3 |
| 26 | Heterogeneous nuclear ribonucleoprotein U OS=Homo sapiens OX=9606 GN=HNRNPU PE=1 SV=6 |
| 27 | Sodium/potassium-transporting ATPase subunit beta-1 OS=Homo sapiens OX=9606 GN=ATP1B1 PE=1 SV=1 |
| 28 | Calumenin OS=Homo sapiens OX=9606 GN=CALU PE=1 SV=2 |
| 29 | Eukaryotic translation initiation factor 3 subunit H OS=Homo sapiens OX=9606 GN=EIF3H PE=1 SV=1 |
| 30 | Phosphoenolpyruvate carboxykinase [GTP], mitochondrial OS=Homo sapiens OX=9606 GN=PCK2 PE=1 SV=4 |
| 31 | Disks large homolog 1 OS=Homo sapiens OX=9606 GN=DLG1 PE=1 SV=2 |
| 32 | E3 ubiquitin-protein ligase NEDD4-1 OS=Homo sapiens OX=9606 GN=NEDD4 PE=1 SV=4 |
| 33 | Golgi-resident adenosine 3',5'-bisphosphate 3'-phosphatase OS=Homo sapiens OX=9606 GN=BPNT2 PE=1 SV=1 |
| 34 | Transmembrane protein 214 OS=Homo sapiens OX=9606 GN=TMEM214 PE=1 SV=2 |
| 35 | Exportin-1 OS=Homo sapiens OX=9606 GN=XPO1 PE=1 SV=1 |
| 36 | Glycosyltransferase 8 domain-containing protein 1 OS=Homo sapiens OX=9606 GN=GLT8D1 PE=1 SV=2 |
| 37 | Disks large homolog 3 OS=Homo sapiens OX=9606 GN=DLG3 PE=1 SV=2 |
| 38 | Acyl-coenzyme A thioesterase 9, mitochondrial OS=Homo sapiens OX=9606 GN=ACOT9 PE=1 SV=2 |
| 39 | Histone-lysine N-methyltransferase SMYD3 OS=Homo sapiens OX=9606 GN=SMYD3 PE=1 SV=4 |
| 40 | Heterogeneous nuclear ribonucleoprotein F OS=Homo sapiens OX=9606 GN=HNRNPF PE=1 SV=3 |
| 41 | Dolichyl-diphosphooligosaccharide--protein glycosyltransferase subunit 1 OS=Homo sapiens OX=9606 GN=RPN1 PE=1 SV=1 |
| 42 | EH domain-containing protein 1 OS=Homo sapiens OX=9606 GN=EHD1 PE=1 SV=2 |
| 43 | ATP-dependent RNA helicase A OS=Homo sapiens OX=9606 GN=DHX9 PE=1 SV=4 |
| 44 | Protein YIPF2 OS=Homo sapiens OX=9606 GN=YIPF2 PE=1 SV=1 |
| 45 | T-complex protein 1 subunit eta OS=Homo sapiens OX=9606 GN=CCT7 PE=1 SV=2 |
| 46 | Splicing factor 1 OS=Homo sapiens OX=9606 GN=SF1 PE=1 SV=4 |
| 47 | Rho guanine nucleotide exchange factor 2 OS=Homo sapiens OX=9606 GN=ARHGEF2 PE=1 SV=4 |
| 48 | Ras GTPase-activating-like protein IQGAP1 OS=Homo sapiens OX=9606 GN=IQGAP1 PE=1 SV=1 |
| 49 | Ribonucleoprotein PTB-binding 1 OS=Homo sapiens OX=9606 GN=RAVER1 PE=1 SV=1 |
| 50 | N-acylneuraminate cytidylyltransferase OS=Homo sapiens OX=9606 GN=CMAS PE=1 SV=2 |
| 51 | Mannosyl-oligosaccharide glucosidase OS=Homo sapiens OX=9606 GN=MOGS PE=1 SV=5 |
| 52 | Ornithine aminotransferase, mitochondrial OS=Homo sapiens OX=9606 GN=OAT PE=1 SV=1 |
| 53 | Zinc finger CCCH-type antiviral protein 1 OS=Homo sapiens OX=9606 GN=ZC3HAV1 PE=1 SV=3 |
| 54 | E3 ubiquitin-protein ligase CBL OS=Homo sapiens OX=9606 GN=CBL PE=1 SV=2 |
| 55 | E3 UFM1-protein ligase 1 OS=Homo sapiens OX=9606 GN=UFL1 PE=1 SV=2 |
| 56 | Transcription intermediary factor 1-beta OS=Homo sapiens OX=9606 GN=TRIM28 PE=1 SV=5 |
| 57 | Plectin OS=Homo sapiens OX=9606 GN=PLEC PE=1 SV=3 |
| 58 | Sorting nexin-9 OS=Homo sapiens OX=9606 GN=SNX9 PE=1 SV=1 |
| 59 | Serine/threonine-protein phosphatase 2A 56 kDa regulatory subunit gamma isoform OS=Homo sapiens OX=9606 GN=PPP2R5C PE=1 SV=3 |
| 60 | Junction-mediating and -regulatory protein OS=Homo sapiens OX=9606 GN=JMY PE=1 SV=2 |
| 61 | ATP-dependent 6-phosphofructokinase, platelet type OS=Homo sapiens OX=9606 GN=PFKP PE=1 SV=2 |
| 62 | Exocyst complex component 4 OS=Homo sapiens OX=9606 GN=EXOC4 PE=1 SV=1 |
| 63 | Tudor domain-containing protein 3 OS=Homo sapiens OX=9606 GN=TDRD3 PE=1 SV=1 |
| 64 | Guanine nucleotide-binding protein-like 3 OS=Homo sapiens OX=9606 GN=GNL3 PE=1 SV=2 |
| 65 | Double-stranded RNA-binding protein Staufen homolog 2 OS=Homo sapiens OX=9606 GN=STAU2 PE=1 SV=2 |
| 66 | Phosphatidate phosphatase LPIN1 OS=Homo sapiens OX=9606 GN=LPIN1 PE=1 SV=2 |
| 67 | Protein salvador homolog 1 OS=Homo sapiens OX=9606 GN=SAV1 PE=1 SV=2 |
| 68 | T-box brain protein 1 OS=Homo sapiens OX=9606 GN=TBR1 PE=1 SV=1 |
| 69 | Cellular tumor antigen p53 OS=Homo sapiens OX=9606 GN=TP53 PE=1 SV=4 |
| 70 | Rab11 family-interacting protein 5 OS=Homo sapiens OX=9606 GN=RAB11FIP5 PE=1 SV=1 |
| 71 | Staphylococcal nuclease domain-containing protein 1 OS=Homo sapiens OX=9606 GN=SND1 PE=1 SV=1 |
| 72 | Apoptosis inhibitor 5 OS=Homo sapiens OX=9606 GN=API5 PE=1 SV=3 |
| 73 | LEM domain-containing protein 2 OS=Homo sapiens OX=9606 GN=LEMD2 PE=1 SV=1 |
| 74 | Cytochrome b-c1 complex subunit 2, mitochondrial OS=Homo sapiens OX=9606 GN=UQCRC2 PE=1 SV=3 |
| 75 | Ral GTPase-activating protein subunit beta OS=Homo sapiens OX=9606 GN=RALGAPB PE=1 SV=1 |
| 76 | La-related protein 4B OS=Homo sapiens OX=9606 GN=LARP4B PE=1 SV=3 |
| 77 | SRSF protein kinase 2 OS=Homo sapiens OX=9606 GN=SRPK2 PE=1 SV=3 |
| 78 | Tubulin beta-6 chain OS=Homo sapiens OX=9606 GN=TUBB6 PE=1 SV=1 |
| 79 | DNA mismatch repair protein Msh6 OS=Homo sapiens OX=9606 GN=MSH6 PE=1 SV=2 |
| 80 | Laminin subunit beta-3 OS=Homo sapiens OX=9606 GN=LAMB3 PE=1 SV=1 |
| 81 | Zinc finger protein 787 OS=Homo sapiens OX=9606 GN=ZNF787 PE=1 SV=4 |
| 82 | ATP-binding cassette sub-family D member 3 OS=Homo sapiens OX=9606 GN=ABCD3 PE=1 SV=1 |
| 83 | Activating signal cointegrator 1 complex subunit 2 OS=Homo sapiens OX=9606 GN=ASCC2 PE=1 SV=3 |
| 84 | Protein SPT2 homolog OS=Homo sapiens OX=9606 GN=SPTY2D1 PE=1 SV=3 |
| 85 | Nuclear export mediator factor NEMF OS=Homo sapiens OX=9606 GN=NEMF PE=1 SV=4 |
| 86 | Aurora kinase A OS=Homo sapiens OX=9606 GN=AURKA PE=1 SV=2 |
| 87 | Developmentally-regulated GTP-binding protein 1 OS=Homo sapiens OX=9606 GN=DRG1 PE=1 SV=1 |
| 88 | Protein PRRC2C OS=Homo sapiens OX=9606 GN=PRRC2C PE=1 SV=4 |
| 89 | Probable ATP-dependent RNA helicase DDX10 OS=Homo sapiens OX=9606 GN=DDX10 PE=1 SV=2 |
| 90 | Coronin-1C OS=Homo sapiens OX=9606 GN=CORO1C PE=1 SV=1 |
| 91 | Mitochondrial proton/calcium exchanger protein OS=Homo sapiens OX=9606 GN=LETM1 PE=1 SV=1 |
| 92 | AP-3 complex subunit mu-1 OS=Homo sapiens OX=9606 GN=AP3M1 PE=1 SV=1 |
| 93 | Caspase-4 OS=Homo sapiens OX=9606 GN=CASP4 PE=1 SV=1 |
| 94 | Synaptic functional regulator FMR1 OS=Homo sapiens OX=9606 GN=FMR1 PE=1 SV=1 |
| 95 | Prolactin regulatory element-binding protein OS=Homo sapiens OX=9606 GN=PREB PE=1 SV=2 |
| 96 | Constitutive coactivator of PPAR-gamma-like protein 1 OS=Homo sapiens OX=9606 GN=FAM120A PE=1 SV=2 |
| 97 | 26S proteasome regulatory subunit 8 OS=Homo sapiens OX=9606 GN=PSMC5 PE=1 SV=1 |
| 98 | Tubulin gamma-1 chain OS=Homo sapiens OX=9606 GN=TUBG1 PE=1 SV=2 |
| 99 | ATP-binding cassette sub-family F member 1 OS=Homo sapiens OX=9606 GN=ABCF1 PE=1 SV=2 |
| 100 | Protein PAT1 homolog 1 OS=Homo sapiens OX=9606 GN=PATL1 PE=1 SV=2 |
| 101 | Ataxin-10 OS=Homo sapiens OX=9606 GN=ATXN10 PE=1 SV=1 |
| 102 | ATP synthase subunit f, mitochondrial OS=Homo sapiens OX=9606 GN=ATP5MF PE=1 SV=3 |
| 103 | WW domain-binding protein 11 OS=Homo sapiens OX=9606 GN=WBP11 PE=1 SV=1 |
| 104 | Eukaryotic translation initiation factor 3 subunit M OS=Homo sapiens OX=9606 GN=EIF3M PE=1 SV=1 |
| 105 | Isocitrate dehydrogenase [NAD] subunit beta, mitochondrial OS=Homo sapiens OX=9606 GN=IDH3B PE=1 SV=2 |
| 106 | Eukaryotic translation initiation factor 3 subunit E OS=Homo sapiens OX=9606 GN=EIF3E PE=1 SV=1 |
| 107 | RNA-binding protein 26 OS=Homo sapiens OX=9606 GN=RBM26 PE=1 SV=3 |
| 108 | Lysophosphatidylserine lipase ABHD12 OS=Homo sapiens OX=9606 GN=ABHD12 PE=1 SV=2 |
| 109 | [Pyruvate dehydrogenase (acetyl-transferring)] kinase isozyme 3, mitochondrial OS=Homo sapiens OX=9606 GN=PDK3 PE=1 SV=1 |
| 110 | Signal transducer and activator of transcription 3 OS=Homo sapiens OX=9606 GN=STAT3 PE=1 SV=2 |
| 111 | Probable ATP-dependent RNA helicase DDX20 OS=Homo sapiens OX=9606 GN=DDX20 PE=1 SV=2 |
| 112 | Trifunctional enzyme subunit beta, mitochondrial OS=Homo sapiens OX=9606 GN=HADHB PE=1 SV=3 |
| 113 | Ras-associated and pleckstrin homology domains-containing protein 1 OS=Homo sapiens OX=9606 GN=RAPH1 PE=1 SV=3 |
| 114 | Kinesin-like protein KIF2A OS=Homo sapiens OX=9606 GN=KIF2A PE=1 SV=3 |
| 115 | Cell division cycle 5-like protein OS=Homo sapiens OX=9606 GN=CDC5L PE=1 SV=2 |
| 116 | PC4 and SFRS1-interacting protein OS=Homo sapiens OX=9606 GN=PSIP1 PE=1 SV=1 |
| 117 | Fragile X mental retardation syndrome-related protein 1 OS=Homo sapiens OX=9606 GN=FXR1 PE=1 SV=3 |
| 118 | Serine/threonine-protein kinase 3 OS=Homo sapiens OX=9606 GN=STK3 PE=1 SV=2 |
| 119 | YTH domain-containing family protein 2 OS=Homo sapiens OX=9606 GN=YTHDF2 PE=1 SV=2 |
| 120 | Lactadherin OS=Homo sapiens OX=9606 GN=MFGE8 PE=1 SV=3 |
| 121 | PDZ and LIM domain protein 7 OS=Homo sapiens OX=9606 GN=PDLIM7 PE=1 SV=1 |
| 122 | Transcriptional activator protein Pur-alpha OS=Homo sapiens OX=9606 GN=PURA PE=1 SV=2 |
| 123 | Splicing factor U2AF 65 kDa subunit OS=Homo sapiens OX=9606 GN=U2AF2 PE=1 SV=4 |
| 124 | Serine/threonine-protein kinase N2 OS=Homo sapiens OX=9606 GN=PKN2 PE=1 SV=1 |
| 125 | Nuclear migration protein nudC OS=Homo sapiens OX=9606 GN=NUDC PE=1 SV=1 |
| 126 | Transducin beta-like protein 2 OS=Homo sapiens OX=9606 GN=TBL2 PE=1 SV=1 |
| 127 | E3 ubiquitin-protein ligase TRIM4 OS=Homo sapiens OX=9606 GN=TRIM4 PE=1 SV=2 |
| 128 | Rho guanine nucleotide exchange factor 12 OS=Homo sapiens OX=9606 GN=ARHGEF12 PE=1 SV=1 |
| 129 | Testin OS=Homo sapiens OX=9606 GN=TES PE=1 SV=1 |
| 130 | ARF GTPase-activating protein GIT1 OS=Homo sapiens OX=9606 GN=GIT1 PE=1 SV=2 |
| 131 | Dolichyl-diphosphooligosaccharide--protein glycosyltransferase 48 kDa subunit OS=Homo sapiens OX=9606 GN=DDOST PE=1 SV=4 |
| 132 | Basic leucine zipper and W2 domain-containing protein 1 OS=Homo sapiens OX=9606 GN=BZW1 PE=1 SV=1 |
| 133 | DNA polymerase delta subunit 3 OS=Homo sapiens OX=9606 GN=POLD3 PE=1 SV=2 |
| 134 | Translocation protein SEC63 homolog OS=Homo sapiens OX=9606 GN=SEC63 PE=1 SV=2 |
| 135 | Cytoskeleton-associated protein 5 OS=Homo sapiens OX=9606 GN=CKAP5 PE=1 SV=3 |
| 136 | Ubiquitin carboxyl-terminal hydrolase 10 OS=Homo sapiens OX=9606 GN=USP10 PE=1 SV=2 |
| 137 | 26S proteasome regulatory subunit 10B OS=Homo sapiens OX=9606 GN=PSMC6 PE=1 SV=1 |
| 138 | AP-3 complex subunit beta-1 OS=Homo sapiens OX=9606 GN=AP3B1 PE=1 SV=3 |
| 139 | Nicotinamide phosphoribosyltransferase OS=Homo sapiens OX=9606 GN=NAMPT PE=1 SV=1 |
| 140 | Eukaryotic translation initiation factor 4 gamma 1 OS=Homo sapiens OX=9606 GN=EIF4G1 PE=1 SV=4 |
| 141 | Eukaryotic initiation factor 4A-III OS=Homo sapiens OX=9606 GN=EIF4A3 PE=1 SV=4 |
| 142 | Cytohesin-3 OS=Homo sapiens OX=9606 GN=CYTH3 PE=1 SV=2 |
| 143 | Dual specificity mitogen-activated protein kinase kinase 2 OS=Homo sapiens OX=9606 GN=MAP2K2 PE=1 SV=1 |
| 144 | Tyrosine--tRNA ligase, mitochondrial OS=Homo sapiens OX=9606 GN=YARS2 PE=1 SV=2 |
| 145 | Isoleucine--tRNA ligase, mitochondrial OS=Homo sapiens OX=9606 GN=IARS2 PE=1 SV=2 |
| 146 | Pumilio homolog 1 OS=Homo sapiens OX=9606 GN=PUM1 PE=1 SV=3 |
| 147 | Lipoma-preferred partner OS=Homo sapiens OX=9606 GN=LPP PE=1 SV=1 |
| 148 | Helicase-like transcription factor OS=Homo sapiens OX=9606 GN=HLTF PE=1 SV=2 |
| 149 | Cysteine desulfurase, mitochondrial OS=Homo sapiens OX=9606 GN=NFS1 PE=1 SV=3 |
| 150 | Probable ATP-dependent RNA helicase DDX17 OS=Homo sapiens OX=9606 GN=DDX17 PE=1 SV=2 |
| 151 | 26S proteasome non-ATPase regulatory subunit 11 OS=Homo sapiens OX=9606 GN=PSMD11 PE=1 SV=3 |
| 152 | Eukaryotic translation initiation factor 2 subunit 3 OS=Homo sapiens OX=9606 GN=EIF2S3 PE=1 SV=3 |
| 153 | 26S proteasome regulatory subunit 6A OS=Homo sapiens OX=9606 GN=PSMC3 PE=1 SV=3 |
| 154 | Protein pelota homolog OS=Homo sapiens OX=9606 GN=PELO PE=1 SV=2 |
| 155 | Armadillo repeat-containing protein 6 OS=Homo sapiens OX=9606 GN=ARMC6 PE=1 SV=2 |
| 156 | Isoleucine--tRNA ligase, cytoplasmic OS=Homo sapiens OX=9606 GN=IARS1 PE=1 SV=2 |
| 157 | Sorting nexin-8 OS=Homo sapiens OX=9606 GN=SNX8 PE=1 SV=1 |
| 158 | General transcription factor IIF subunit 1 OS=Homo sapiens OX=9606 GN=GTF2F1 PE=1 SV=2 |
| 159 | Acylglycerol kinase, mitochondrial OS=Homo sapiens OX=9606 GN=AGK PE=1 SV=2 |
| 160 | Melanoma-associated antigen D2 OS=Homo sapiens OX=9606 GN=MAGED2 PE=1 SV=2 |
| 161 | Filamin-A OS=Homo sapiens OX=9606 GN=FLNA PE=1 SV=4 |
| 162 | Liprin-alpha-1 OS=Homo sapiens OX=9606 GN=PPFIA1 PE=1 SV=1 |
| 163 | Aspartate--tRNA ligase, cytoplasmic OS=Homo sapiens OX=9606 GN=DARS1 PE=1 SV=2 |
| 164 | Interferon regulatory factor 2-binding protein 1 OS=Homo sapiens OX=9606 GN=IRF2BP1 PE=1 SV=1 |
| 165 | T-complex protein 1 subunit beta OS=Homo sapiens OX=9606 GN=CCT2 PE=1 SV=4 |
| 166 | Probable ATP-dependent RNA helicase DDX6 OS=Homo sapiens OX=9606 GN=DDX6 PE=1 SV=2 |
| 167 | Moesin OS=Homo sapiens OX=9606 GN=MSN PE=1 SV=3 |
| 168 | Glutaryl-CoA dehydrogenase, mitochondrial OS=Homo sapiens OX=9606 GN=GCDH PE=1 SV=1 |
| 169 | Polyadenylate-binding protein 4 OS=Homo sapiens OX=9606 GN=PABPC4 PE=1 SV=1 |
| 170 | Saccharopine dehydrogenase-like oxidoreductase OS=Homo sapiens OX=9606 GN=SCCPDH PE=1 SV=1 |
| 171 | NADH dehydrogenase [ubiquinone] iron-sulfur protein 2, mitochondrial OS=Homo sapiens OX=9606 GN=NDUFS2 PE=1 SV=2 |
| 172 | Actin, alpha cardiac muscle 1 OS=Homo sapiens OX=9606 GN=ACTC1 PE=1 SV=1 |
| 173 | WD repeat-containing protein 41 OS=Homo sapiens OX=9606 GN=WDR41 PE=1 SV=3 |
| 174 | HLA class I histocompatibility antigen, A alpha chain OS=Homo sapiens OX=9606 GN=HLA-A PE=1 SV=2 |
| 175 | Exocyst complex component 7 OS=Homo sapiens OX=9606 GN=EXOC7 PE=1 SV=3 |
| 176 | LanC-like protein 2 OS=Homo sapiens OX=9606 GN=LANCL2 PE=1 SV=1 |
| 177 | DDRGK domain-containing protein 1 OS=Homo sapiens OX=9606 GN=DDRGK1 PE=1 SV=2 |
| 178 | Proteasome adapter and scaffold protein ECM29 OS=Homo sapiens OX=9606 GN=ECPAS PE=1 SV=2 |
| 179 | Cell growth-regulating nucleolar protein OS=Homo sapiens OX=9606 GN=LYAR PE=1 SV=2 |
| 180 | ATP-dependent RNA helicase DDX3X OS=Homo sapiens OX=9606 GN=DDX3X PE=1 SV=3 |
| 181 | NFATC2-interacting protein OS=Homo sapiens OX=9606 GN=NFATC2IP PE=1 SV=1 |
| 182 | PCI domain-containing protein 2 OS=Homo sapiens OX=9606 GN=PCID2 PE=1 SV=2 |
| 183 | Headcase protein homolog OS=Homo sapiens OX=9606 GN=HECA PE=1 SV=1 |
| 184 | 2'-5'-oligoadenylate synthase 3 OS=Homo sapiens OX=9606 GN=OAS3 PE=1 SV=3 |
| 185 | Transmembrane protein 43 OS=Homo sapiens OX=9606 GN=TMEM43 PE=1 SV=1 |
| 186 | RNA-binding protein 39 OS=Homo sapiens OX=9606 GN=RBM39 PE=1 SV=2 |
| 187 | Regulator of nonsense transcripts 1 OS=Homo sapiens OX=9606 GN=UPF1 PE=1 SV=2 |
| 188 | LIM and calponin homology domains-containing protein 1 OS=Homo sapiens OX=9606 GN=LIMCH1 PE=1 SV=4 |
| 189 | RNA-binding protein NOB1 OS=Homo sapiens OX=9606 GN=NOB1 PE=1 SV=1 |
| 190 | Calcium-binding mitochondrial carrier protein SCaMC-1 OS=Homo sapiens OX=9606 GN=SLC25A24 PE=1 SV=2 |
| 191 | Tumor necrosis factor alpha-induced protein 2 OS=Homo sapiens OX=9606 GN=TNFAIP2 PE=1 SV=2 |
| 192 | Methylthioribose-1-phosphate isomerase OS=Homo sapiens OX=9606 GN=MRI1 PE=1 SV=1 |
| 193 | CAD protein OS=Homo sapiens OX=9606 GN=CAD PE=1 SV=3 |
| 194 | Clathrin heavy chain 1 OS=Homo sapiens OX=9606 GN=CLTC PE=1 SV=5 |
| 195 | C-1-tetrahydrofolate synthase, cytoplasmic OS=Homo sapiens OX=9606 GN=MTHFD1 PE=1 SV=3 |
| 196 | CTTNBP2 N-terminal-like protein OS=Homo sapiens OX=9606 GN=CTTNBP2NL PE=1 SV=2 |
| 197 | Sulfide:quinone oxidoreductase, mitochondrial OS=Homo sapiens OX=9606 GN=SQOR PE=1 SV=1 |
| 198 | WD repeat-containing protein 11 OS=Homo sapiens OX=9606 GN=WDR11 PE=1 SV=1 |
| 199 | FAS-associated factor 2 OS=Homo sapiens OX=9606 GN=FAF2 PE=1 SV=2 |
| 200 | Eukaryotic translation initiation factor 3 subunit F OS=Homo sapiens OX=9606 GN=EIF3F PE=1 SV=1 |
| 201 | ATP-dependent 6-phosphofructokinase, liver type OS=Homo sapiens OX=9606 GN=PFKL PE=1 SV=6 |
| 202 | Cold shock domain-containing protein E1 OS=Homo sapiens OX=9606 GN=CSDE1 PE=1 SV=2 |
| 203 | 2',3'-cyclic-nucleotide 3'-phosphodiesterase OS=Homo sapiens OX=9606 GN=CNP PE=1 SV=2 |
| 204 | Heparan sulfate 2-O-sulfotransferase 1 OS=Homo sapiens OX=9606 GN=HS2ST1 PE=1 SV=1 |
| 205 | Exocyst complex component 1 OS=Homo sapiens OX=9606 GN=EXOC1 PE=1 SV=4 |

**Table S5** Hematological parameters for female NOD/SCID mice.

The data are shown as the mean ± SD (*n* = 5).

| Hematological parameters | PGC-1α^WT^ | PGC-1α^WT^  + Shikonin | PGC-1α^Α295^ | PGC-1α^Α295^  +Shikonin |
| --- | --- | --- | --- | --- |
| White blood cells (×10^9^/L) | 1.46 ± 0.42 | 1.92 ± 0.31 | 1.32 ± 0.46 | 1.50 ± 0.48 |
| Lymphocytes (×10^9^/L) | 0.44 ± 0.10 | 0.58 ± 0.10 | 0.40 ± 0.14 | 0.52 ± 0.07 |
| Monocytes (×10^9^/L) | 0.06 ± 0.05 | 0.12 ± 0.04 | 0.10 ± 0.00 | 0.08 ± 0.07 |
| Granulocytes (×10^9^/L) | 0.96 ± 0.37 | 1.22 ± 0.26 | 0.82 ± 0.33 | 0.90 ± 0.35 |
| Red blood cells (×10^12^/L) | 8.77 ± 0.98 | 9.36 ± 0.09 | 8.69 ± 1.19 | 9.39 ± 0.22 |
| Hemoglobin (g/L) | 147.8 ± 6.5 | 153.0 ± 4.6 | 146.8 ± 6.1 | 154.4 ± 3.4 |
| Hematocrit (%) | 49.82 ± 4.72 | 50.90 ± 2.93 | 49.78 ± 4.92 | 48.56 ± 4.95 |
| Mean corpuscular volume (fL) | 56.92 ± 1.38 | 54.36 ± 3.12 | 57.66 ± 2.84 | 51.72 ± 5.14 |
| Mean corpuscular hemoglobin (pg) | 17.10 ± 2.40 | 16.34 ± 0.58 | 17.18 ± 2.18 | 16.46 ± 0.58 |
| Mean corpuscular hemoglobin concentration (g/L) | 300.0 ± 36.1 | 302.2 ± 26.0 | 297.2 ± 23.4 | 322.0 ± 41.9 |
| Red blood cell distribution width (%) | 17.02 ± 0.29 | 17.44 ± 0.39 | 16.96 ± 0.30 | 16.66 ± 0.63 |
| Platelet (×10^9^/L) | 1459.6 ± 112.1 | 1593.2 ± 93.2 | 1417.2 ± 146.8 | 1519.4 ± 93.2 |
| Mean platelet volume (fL) | 5.92 ± 0.26 | 6.22 ± 0.56 | 6.36 ± 0.60 | 5.80 ± 0.14 |

**Table S6** Functional parameters of heart, liver and kidney in NOD/SCID mice.

| Treatment Group | PGC-1α^WT^ | PGC-1α^WT^  + Shikonin | PGC-1α^Α295^ | PGC-1α^Α295^  +Shikonin |
| --- | --- | --- | --- | --- |
| Heart Markers (Blood sample) | | | |  |
| Lactic dehydrogenase  (LDH, U/L) | 210.28 ± 24.98 | 188.73 ± 13.78 | 189.36 ± 11.94 | 198.95 ± 22.11 |
| Creatine kinase (CK, U/L) | 39.89 ± 1.33 | 39.76 ± 2.02 | 41.14 ± 2.24 | 39.69 ± 1.10 |
| Liver Markers (Blood sample) | | | |  |
| Albumin (ALB, g/L) | 28.16 ± 0.30 | 27.33 ± 1.32 | 27.39 ± 1.32 | 29.70 ± 2.31 |
| Transaminase (ALT, IU/L) | 46.61 ± 2.44 | 45.56 ± 2.18 | 44.88 ± 1.00 | 45.55 ± 1.45 |
| Aspartate aminotransferase (AST, IU/L) | 126.87 ± 13.06 | 133.58 ± 16.52 | 144.82 ± 11.72 | 119.51 ± 25.06 |
| Kidney Markers (Blood sample) | | | |  |
| Blood Urea Nitrogen  (BUN, mM/L) | 13.30 ± 0.83 | 12.92 ± 1.14 | 12.49 ± 1.08 | 13.28 ± 1.24 |
| Serum Creatinine  (Src, μM/L) | 22.15 ± 4.25 | 17.40 ± 1.69 | 19.71 ± 2.09 | 19.92 ± 2.94 |
| Uric Acid (UA, μM/L) | 96.82 ± 5.82 | 104.71 ± 7.48 | 94.52 ± 8.49 | 98.47 ± 5.82 |

The data are shown as the mean ± SD (*n* = 5).
